# Spike-based time-domain analog weighted-sum calculation model for extremely low power VLSI implementation of multi-layer neural networks

**DOI:** 10.3389/fnins.2025.1656892

**Published:** 2025-09-12

**Authors:** Quan Wang, Hakaru Tamukoh, Takashi Morie

**Affiliations:** ^1^Graduate School of Life Science and Systems Engineering, Kyushu Institute of Technology, Kitakyushu, Japan; ^2^Research Center for Neuromorphic AI Hardware, Kyushu Institute of Technology, Kitakyushu, Japan

**Keywords:** time-domain analog computing, weighted sum, spike-based computing, deep neural networks, multi-layer perceptron, artificial intelligence hardware, AI processor, matrix-vector multiplication

## Abstract

In deep neural network (DNN) models, the weighted summation, or multiply-and-accumulate (MAC) operation, is an essential and heavy calculation task, which leads to high power consumption in current digital processors. The use of analog operation in complementary metal-oxide-semiconductor (CMOS) very-large-scale integration (VLSI) circuits is a promising method for achieving extremely low power-consumption operation for such calculation tasks. In this paper, a time-domain analog weighted-sum calculation model is proposed based on an integrate-and-fire-type spiking neuron model. The proposed calculation model is applied to multi-layer feedforward networks, in which weighted summations with positive and negative weights are separately performed, and two timings proportional to the positive and negative ones are produced, respectively, in each layer. The timings are then fed into the next layers without their subtraction operation. We also propose VLSI circuits to implement the proposed model. Unlike conventional analog voltage or current mode circuits, the time-domain analog circuits use transient operation in charging/discharging processes to capacitors. Since the circuits can be designed without operational amplifiers, they can operate with extremely low power consumption. We designed a proof-of-concept (PoC) CMOS circuit to verify weighted-sum operation with the same weights. Simulation results showed that the precision was above 4-bit, and the energy efficiency for the weighted-sum calculation was 237.7 Tera Operations Per Second Per Watt (TOPS/W), more than one order of magnitude higher than that in state-of-the-art digital AI processors. Our model promises to be a suitable approach for performing intensive in-memory computing (IMC) of DNNs with moderate precision very energy-efficiently while reducing the cost of analog-digital-converter (ADC) overhead.

## 1 Introduction

Artificial neural networks (ANNs) or deep neural networks (DNNs), such as convolutional deep neural networks (CNNs) (LeCun et al., 2002) and fully connected multi-layer perceptrons (MLPs) (Cireşan et al., 2010), have shown excellent performance on various intelligent tasks, such as object detection and image classification (Cireşan et al., 2010; Krizhevsky et al., 2012; LeCun et al., 2015). However, DNNs require an enormous number of parameters and computational capability, resulting in much heavier computation and data movements, which leads to high power consumption in current digital computers, and even in highly parallel coprocessors such as graphics processing units (GPUs). To implement ANNs at edge devices such as mobile phones and personal service robots, very low power consumption operation is required.

In ANN models, the weighted summation, or multiply-and-accumulate (MAC) operation, is an essential and heavy calculation task (Shukla et al., 2019), and dedicated complementary metal-oxide-semiconductor (CMOS) very-large-scale integration (VLSI) processors have been developed to accomplish it (Chen et al., 2020). As an implementation approach other than digital processors, the use of analog operation in CMOS VLSI circuits is a promising method for achieving extremely low power-consumption operation for such calculation tasks (Hasler and Marr, 2013; Fick et al., 2017; Mahmoodi and Strukov, 2018; Bavandpour et al., 2020).

From the perspective of computing architecture, it is well known that traditional von Neumann-based architectures require the movement of weights and intermediate computing results between memory and processing units, resulting in extra latency and energy consumption, which is further aggravated in the data-intensive applications of DNNs (Horowitz, 2014; Sze et al., 2017). To reduce or eliminate power consumption and latency of data movement over memory, in-memory computing (IMC) with SRAM or memristive devices, which act as analog memory, is becoming a promising paradigm for accelerating DNNs. Analog in-memory computing (AIMC), which combines analog computation with the IMC architecture, can provide better energy efficiency by performing MACs in parallel-also known as matrix-vector multiplications (MVMs)-within the memory array in a single step (Verma et al., 2019; Valavi et al., 2019; Jiang et al., 2020; Jia et al., 2021a; Prezioso et al., 2015; Shafiee et al., 2016; Tsai et al., 2018; Khaddam-Aljameh et al., 2022). Typically, an AIMC core executes analog MVMs for a single ANN layer, multiplying the stationary weight matrix stored in the core with the activation vector applied at its input. SRAM-based implementations cannot hold the whole weight of larger networks fully on-chip because of their large areas for storing multi-bit weights (Jia et al., 2021b). As a result, off-chip weight buffers are needed to store network weights and transfer them partially to AIMC cores, which further reduces energy efficiency. Additionally, SRAM's volatility means that on-chip weights are lost when power is turned off. Analog non-volatile memory (NVM) technologies, such as resistive memory and flash memory, offer multiple bits per device, high density, and non-volatility, making it possible to store entire network weights on-chip. This has led to an emerging trend in NVM-based analog AI systems, where an increasing number of AIMC cores or tiles are deployed to efficiently conduct inference tasks (Wan et al., 2022; Fick et al., 2022; Le Gallo et al., 2023; Ambrogio et al., 2023).

Despite the exciting opportunities for energy-efficient ANN processing, AIMC-based AI systems also present unique challenges that must be addressed to realize their full potential. Beyond the limitations in computation accuracy, a critical challenge is the additional need for digital-to-analog converters (DACs) and analog-to-digital converters (ADCs) to transfer intermediate data between layers or tiles in digital form and to interface with digital peripheral circuits when MAC computations are performed in the current or voltage domain. These converters significantly limit the energy efficiency and scalability of AIMC systems (Shafiee et al., 2016; Marinella et al., 2018; Tsai et al., 2018; Verma et al., 2019; Jia et al., 2021a). To mitigate or overcome the limitations of DACs and ADCs, AIMC cores are increasingly adopting time-domain (TD) computing for MAC operations and interfacing with digital systems through digital-to-time converters (DTCs) and time-to-digital converters (TDCs) (Bavandpour et al., 2019a,b, 2021; Yang et al., 2019; Freye et al., 2022; Wu et al., 2022; Al Maharmeh et al., 2023; Choi et al., 2023). TD computing offers better technology scaling than voltage- and current-domain approaches (Al Maharmeh et al., 2020; Freye et al., 2024; Al Maharmeh et al., 2024), while DTCs and TDCs are generally more energy- and area-efficient than DACs and ADCs (Chen et al., 2022; Khaddam-Aljameh et al., 2022). Most TD schemes represent data using pulse-width modulation (PWM) or delay. In multi-core TD AIMC systems, time-based communication-where pulses are transmitted directly from tile to tile or layer to layer in an analog manner-is emerging as another key trend (Lim et al., 2020; Narayanan et al., 2021; Jiang et al., 2022; Seo et al., 2022; Nägele et al., 2023; Ambrogio et al., 2023). Because of the elimination of most DTCs and TDCs, system energy efficiency can be further improved. However, the TD analog computation is inherently susceptible to analog non-idealities, such as process, voltage, and temperature (PVT) variations, limiting the computation precision. Regarding accuracy, it is now widely recognized that 4-8 bits provide stable inference performance for most mainstream applications (Gupta et al., 2015; McKinstry et al., 2018; Choi et al., 2018), and this level of precision can typically be achieved through carefully designed analog circuits.

Successful DNNs are based on the second-generation artificial neuron model, which processes real-valued data and utilizes nonlinear activation functions (Roy et al., 2019). Most DNN architectures require signed computations. Since the rectified linear unit (ReLU) activation function (Nair and Hinton, 2010) is commonly used and the inputs of the first layer can be normalized as non-negative, IMC architectures have primarily been explored for two-quadrant MACs. However, to accommodate a wider range of applications, the AIMC system needs to support four-quadrant MACs (Khaddam-Aljameh et al., 2022; Le Gallo et al., 2023; Ambrogio et al., 2023; Le Gallo et al., 2024).

The time-domain weighted-sum calculation model was initially proposed based on the third-generation neuron model-spiking neurons inspired by the behavior of biological neurons (Maass, 1997a,b, 1999), to implement real-valued MACs in ANNs. In the model, inputs and outputs are encoded as spike timings, and weights are represented by the rising slope of the post-synaptic potential (PSP).

Subsequent research has simplified and expanded this model under the assumption of operation in analog circuits with transient states (Morie et al., 2016, 2010; Tohara et al., 2016). However, they have been limited to one-quadrant weighted-sum models where all weights for a single neuron share the same sign, and these studies did not address how to apply their models to neural networks. The proposed analog circuit, consisting of multiple input resistive elements and a capacitor (an RC circuit), enables extremely low-power operation, with energy consumption potentially reduced to the order of 0.1 fJ per operation. Throughout this paper, we refer to this VLSI implementation approach as “time-domain analog computing with transient states (TACT).” Unlike conventional weighted-sum operations in analog voltage or current modes, the TACT approach is well-suited for achieving much lower power consumption in CMOS VLSI implementations of ANNs. In this work, we extend the model to address the above-described challenges associated with NVM-based AIMC AI systems. Our primary contributions are summarized as follows.

1) We extend the time-domain one-quadrant MAC calculation model to four-quadrant one, where signed inputs are encoded using a differential pair of spikes, and signed weights are implemented through a dummy weights scheme. The output is represented by a pair of spikes, with their timing difference proportional to the MAC result, enabled by the added dummy weights. Since both inputs and outputs are encoded in a timing format, the AIMC core can be seamlessly integrated with efficient DTCs and TDCs.
2) We theoretically demonstrate how MAC output spikes can be directly transferred to the next layer, potentially eliminating the need for DTCs and TDCs between tiles. Additionally, we provide a clear explanation of the challenges associated with spike transfer in this process.
3) We propose two sets of analog circuits, each consisting of multiple input resistive elements and a capacitor (an RC circuit), to implement the four-quadrant MAC computation. Additionally, we describe a proof-of-concept (PoC) CMOS circuit equivalent to the RC circuit, with a preliminary estimation suggesting that the energy efficiency could reach hundreds of TOPS/W (Tera Operations Per Second Per Watt) and the precision could be four bit or higher.
4) We propose architectures for an analog NVM-based AIMC core and system. In the core, signed weights can be implemented using either a complementary scheme or a differential scheme, depending on how dummy weights are introduced. At the system level, tile-to-tile communication is achieved through spike-based transmission in an analog manner.

## 2 Spike-based time-domain weighted-sum calculation model

### 2.1 Time-domain weighted-sum calculation with same-signed weights

**Figure 1:**
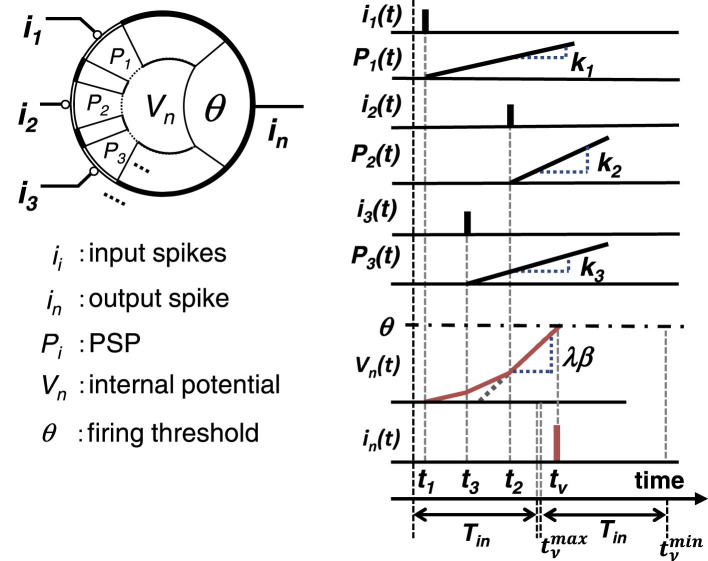
IF neuron model for weighted-sum operation: schematic of the model and weighted-sum operation using the rise timing of PSPs.

A simple spiking neuron model, also known as an integrate-and-fire-type (IF) neuron model, is shown in Figure 1 (Maass, 1999). In this model, a neuron receives spike pulses via synapses. A spike pulse only indicates the input timing, and its pulse width and amplitude do not affect the following processing. A spike generates a temporal voltage change, which is called a post-synaptic potential (PSP), and the internal potential of the *n*-th neuron, *V*_*n*_(*t*), is equal to the spatiotemporal summation of all PSPs. When *V*_*n*_(*t*) reaches the firing threshold θ, the neuron outputs a spike, and *V*_*n*_(*t*) then settles back to the steady state.

Based on the model proposed in Maass (1997a), a simplified weighted-sum operation model using IF neurons is proposed. The time span *T*_*in*_ is defined, during which only one spike is fed from each neuron, and it is assumed that a PSP generated by a spike from neuron *i* increases linearly with slope *k*_*i*_ from the timing of the spike input, *t*_*i*_, as shown in Figure 1.

A required weighted-sum operation is that normalized variables *x*_*i*_ (0 ≤ *x*_*i*_ ≤ 1, *i* = 1, 2, ⋯ , *N*) are multiplied by weight coefficients *a*_*i*_, and the multiplication results are summed regarding *i*, where *N* is the number of inputs. This weighted-sum operation can be performed using the rise timing of PSPs in the IF neuron model. Input spike timing *t*_*i*_ is determined based on *x*_*i*_ using the following relation:


(1) $$\begin{array}{lll} t_{i} & = & T_{in}\left(1-x_{i}\right), \end{array}$$

(2) $$\begin{array}{lll} x_{i} & = & \left(1-\frac{t_{i}}{T_{in}}\right). \end{array}$$

Coefficients *a*_*i*_ are transformed into the PSP slopes *k*_*i*_:


(3) $$\begin{array}{l} k_{i}=\lambda a_{i}, \end{array}$$

where λ is a positive constant. If the firing time of the neuron is defined as *t*_ν_, we easily obtain the equation


(4) $$\begin{array}{l} \overset{N}{\underset{i=1}{\sum }}k_{i}\left(t_{\nu }-t_{i}\right)=\theta . \end{array}$$

If we define the following parameters:


(5) $$\begin{array}{l} \beta =\overset{N}{\underset{i=1}{\sum }}a_{i}, \end{array}$$

we obtain


(6) $$\begin{array}{lll} \overset{N}{\underset{i=1}{\sum }}a_{i}\cdot x_{i} & = & \frac{\theta /\lambda +\beta \left(T_{in}-t_{\nu }\right)}{T_{in}}, \end{array}$$

(7) $$\begin{array}{ll} = & \frac{\theta }{\lambda T_{in}}+\beta \left(1-\frac{t_{\nu }}{T_{in}}\right). \end{array}$$

Here, we assume that all the weights in the calculation have the same sign, i.e., *a*_*i*_≥0 or *a*_*i*_ ≤ 0 for all *i*. This is different from the previous similar work in which only the sum of weights is restricted to be positive for firing (Zhang et al., 2021). When all inputs are minimum (∀*i x*_*i*_ = 0), the left side of Equation 6 is zero. Then, the output timing *t*_ν_ is given by


(8) $$\begin{array}{l} t_{\nu }^{min}=\frac{\theta }{\lambda \beta }+T_{in}. \end{array}$$

On the other hand, when all inputs are maximum (∀*i x*_*i*_ = 1), the left side of Equation 6 is β, and the output timing *t*_ν_ is given by


(9) $$\begin{array}{l} t_{\nu }^{max}=\frac{\theta }{\lambda \beta }. \end{array}$$

The time span during which *t*_ν_ can be output is $\left[t_{\nu }^{max},t_{\nu }^{min}\right]$, and its interval is


(10) $$\begin{array}{l} T_{out}\equiv t_{\nu }^{min}-t_{\nu }^{max}=T_{in}. \end{array}$$

Thus, the time span of output spikes is the same as that of input spikes, *T*_*in*_.

In this model, since the normalization of the sum of *a*_*i*_ (β = 1) is not required [unlike in the previous work (Maass, 1997a, 1999; Tohara et al., 2016)], the calculation process becomes much simpler. When implementing the time-domain weighted-sum operation, setting the threshold potential θ properly is the key to making the operation work appropriately. As shown in Figure 1, the earliest output spike timing has to be later than the latest input spike timing *T*_*in*_; that is, $t_{\nu }^{max}\geq T_{in}$. Thus,


(11) $$\begin{array}{l} \theta \geq \lambda \beta T_{in}. \end{array}$$

Also, we can rewrite Equation 11 as


(12) $$\begin{array}{lll} \theta & = & \lambda \beta T_{in}+\delta , \end{array}$$

(13) $$\begin{array}{lll} \delta & = & \epsilon \left(\lambda \beta T_{in}\right), \end{array}$$

where ϵ≥0 is an arbitrarily small value. By substituting Equations 12, 13 into Equations 8, 9, we obtain


(14) $$\begin{array}{l} t_{\nu }^{min}=\left(2+\epsilon \right)T_{in}, \end{array}$$

(15) $$\begin{array}{l} t_{\nu }^{max}=\left(1+\epsilon \right)T_{in}, \end{array}$$

where ϵ*T*_*in*_ is considered as a time slot between input and output timing spans, as shown in Figure 1, and ϵ determines the length of the slot.

### 2.2 Time-domain weighted-sum calculation with different-signed weights

We propose a time-domain weighted-sum calculation model with two spiking neurons, one for all the positive weights and the other for all the negative ones. We apply Equation 6 to each neuron, and the two results are summed as the final result of the original weighted sum. Here, we show the details of the model.

Let $a_{i}^{+}$ and $a_{i}^{-}$ indicate the positive and negative weights, respectively. We define


(16) $$\begin{array}{l} \beta^{+}=\overset{N^{+}}{\underset{i=1}{\sum }}a_{i}^{+}\geq 0,\;\beta^{-}=\overset{N^{-}}{\underset{i=1}{\sum }}a_{i}^{-}\leq 0. \end{array}$$

Where *N*^+^ and *N*^−^ are the numbers of positive and negative weights, respectively:


(17) $$\begin{array}{l} N=N^{+}+N^{-},\;\overset{N}{\underset{i=1}{\sum }}a_{i}=\overset{N^{+}}{\underset{i=1}{\sum }}a_{i}^{+}+\overset{N^{-}}{\underset{i=1}{\sum }}a_{i}^{-},\;\beta =\beta^{+}+\beta^{-}. \end{array}$$

Thus, assuming λ = 1, Equation 4 is rewritten for the positive and negative weighted-sum operations as


(18) $$\begin{array}{l} \overset{N^{+}}{\underset{i=1}{\sum }}a_{i}^{+}\left(t_{\nu }^{+}-t_{i}\right)=\theta^{+}, \end{array}$$

(19) $$\begin{array}{l} \overset{N^{-}}{\underset{i=1}{\sum }}a_{i}^{-}\left(t_{\nu }^{-}-t_{i}\right)=\theta^{-}, \end{array}$$

where θ^+^(>0), θ^−^(<0), and $t_{\nu }^{+}$ and $t_{\nu }^{-}$ indicate the threshold values and output timings for the positively and negatively weighted-sum operation, respectively. We obtain


(20) $$\begin{array}{lll} \overset{N^{+}}{\underset{i=1}{\sum }}a_{i}^{+}\cdot x_{i} & = & \frac{\theta^{+}+\beta^{+}\left(T_{in}-t_{\nu }^{+}\right)}{T_{in}}, \end{array}$$

(21) $$\begin{array}{lll} \overset{N^{-}}{\underset{i=1}{\sum }}a_{i}^{-}\cdot x_{i} & = & \frac{\theta^{-}+\beta^{-}\left(T_{in}-t_{\nu }^{-}\right)}{T_{in}}. \end{array}$$

Therefore, we can obtain the original weighted-sum result:


(22) $$\begin{array}{l} \sum_{i=1}^{N}a_{i}\cdot x_{i}=\sum_{i=1}^{N^{+}}a_{i}^{+}\cdot x_{i}+\sum_{i=1}^{N^{-}}a_{i}^{-}\cdot x_{i}\\ \;=\frac{\theta^{+}+\theta^{-}+\beta T_{in}-(\beta^{+}t_{\nu }^{+}+\beta^{-}t_{\nu }^{-})}{T_{in}} \end{array}$$

Let us define a dummy weight *a*_0_ as the difference between both absolute values of β^±^:


(23) $$\begin{array}{l} a_{0}=-\left(\beta^{+}+\beta^{-}\right). \end{array}$$

If β^+^≥−β^−^, then *a*_0_ ≤ 0 and this dummy weight is incorporated into the negative weight group, and vice versa. This dummy weight is related to a zero input, *x*_0_ = 0, which means *t*_0_ = *T*_*in*_. By using the dummy weight, we can make the absolute values of β^±^ identical (β = 0), and we define


(24) $$\begin{array}{l} \beta_{o}=\beta^{+}=-\beta^{-}. \end{array}$$

Also, according to Equations 12, 13, the absolute values of θ^+^ and θ^−^ can be the same, and θ^+^+θ^−^ = 0. Therefore, Equation 22 can be rewritten as


(25) $$\begin{array}{l} \overset{N}{\underset{i=1}{\sum }}a_{i}\cdot x_{i}=\frac{\beta_{o}\left(t_{\nu }^{-}-t_{\nu }^{+}\right)}{T_{in}}. \end{array}$$

## 3 Time domain neural network model

### 3.1 Neuron model

**Figure 2:**
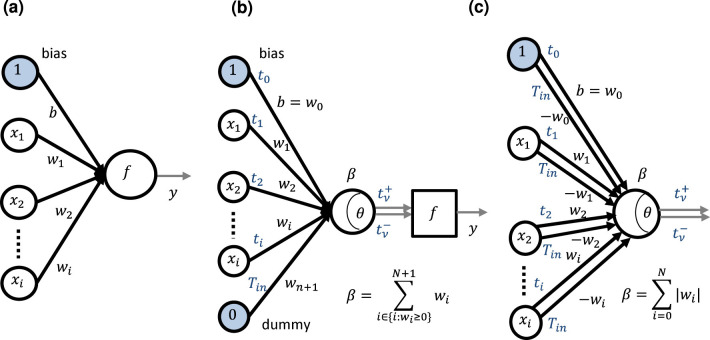
Neuron model: **(a)** typical neuron model; **(b)** neuron model for time-domain weighted-sum operation with a dummy weight, *w*_*n*+1_; **(c)** neuron model for time-domain weighted-sum operation in which each synapse has two sets of inputs and weights that one set is (*x*_*i*_, *w*_*i*_) and the other is (0, −*w*_*i*_) or (*t*_*i*_, *w*_*i*_) and (*T*_*in*_, −*w*_*i*_) according to Equation 2.

The typical neuron model of ANNs is shown in Figure 2a, which has *N* inputs *x*_*i*_ with weights *w*_*i*_ and a bias *b*;


(26) $$\begin{array}{l} y=f\left(\overset{N}{\underset{i=1}{\sum }}w_{i}\cdot x_{i}+b\right), \end{array}$$

where *y* is the output of the neuron, and *f* is an activation function. We can consider the bias as a weight whose input is always unity and regard the 0 index weight as the bias throughout this paper. Therefore, our time-domain weighted-sum calculation model with the dummy weight can be applied to this neuron model, as shown in Figure 2b. According to Equation 25,


(27) $$\begin{array}{l} \overset{N}{\underset{i=0}{\sum }}w_{i}\cdot x_{i}=\frac{\beta \left(t_{\nu }^{-}-t_{\nu }^{+}\right)}{T_{in}}. \end{array}$$

Based on Equation 27, we propose another model, shown in Figure 2c, in which each synapse has two sets of inputs and weights; one is (*x*_*i*_, *w*_*i*_) and the other is (0, −*w*_*i*_). In this model, it is not necessary to add a dummy weight because the summation of positive weights is equal to the absolute one of negative weights automatically, i.e., $\beta =\overset{N}{\underset{i=0}{\sum }}||w_{i}||$.

As the activation function *f*, we often use the rectified linear unit called “ReLU” (Nair and Hinton, 2010), which is defined as follows:


(28) $$f(x)=ReLU(x)=\{\begin{array}{ll} x & if\;x\geq 0,\\ 0 & otherwise \end{array}.$$

We can implement the ReLU function by comparing the output timings $t_{\nu }^{-}$ and $t_{\nu }^{+}$ in the time-domain weighted-sum calculation as follows:


(29) $$\begin{array}{l} f\left(\overset{N}{\underset{i=0}{\sum }}w_{i}\cdot x_{i}\right)=ReLU\left(\frac{\beta \left(t_{\nu }^{-}-t_{\nu }^{+}\right)}{T_{in}}\right)=\frac{\beta \left(t_{\nu }^{-}-t_{\nu }^{+}\right)}{T_{in}} \end{array}$$

where, if $t_{\nu }^{-}>t_{\nu }^{+}$, the difference between the two timing values is regarded as the output transferred to neurons in the next layer, and if $t_{\nu }^{-}<t_{\nu }^{+}$, we set $t_{\nu }^{-}$ and $t_{\nu }^{+}$ to be identical to make the output zero because of the negative weighted-sum result. Its circuit implementation will be shown later.

### 3.2 Neural network model

**Figure 3:**
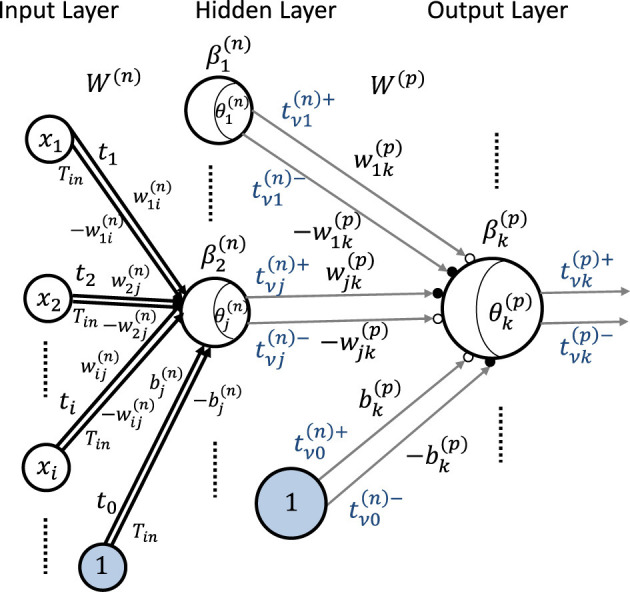
General neural network model with two inputs and outputs for time-domain weighted-sum calculation with positive and negative weights.

In this section, we extend our time-domain neuron model shown in Figure 2c to the neural network and theoretically show the intermediate timing transfer mechanisms between layers. We first apply the procedure to a two-layer MLP, which has one hidden layer and two sets of input and weight for each neuron shown in Figure 3, as an example, and then generalize it.

In this MLP, according to Equation 27, the weighted-sum result of the *j*-th neuron in the hidden layer labeled as *n* can be


(30) $$\begin{array}{l} \overset{N}{\underset{i=0}{\sum }}w_{ij}^{\left(n\right)}\cdot x_{i}=\frac{\beta_{j}^{\left(n\right)}}{T_{in}}\left(t_{vj}^{\left(n\right)-}-t_{vj}^{\left(n\right)+}\right), \end{array}$$

where $\beta_{j}^{\left(n\right)}=\overset{N}{\underset{i=0}{\sum }}||w_{ij}^{\left(n\right)}||$, $t_{vj}^{\left(n\right)-}$ and $t_{vj}^{\left(n\right)+}$ are the timings generated at the *j*-th neuron in the *n*-th layer. The output $y_{k}^{\left(p\right)}$ of the *k*-th neuron in the output layer labeled as *p*(= *n*+1) is


(31) $$\begin{array}{l} y_{k}^{\left(p\right)}=\sum_{j=1}^{N}w_{jk}^{\left(p\right)}\cdot f(\sum_{i=0}^{N}w_{ij}^{\left(n\right)}\cdot x_{i})+b_{k}^{\left(p\right)}\\ \;=\sum_{j=1}^{N}w_{jk}^{\left(p\right)}\cdot f(\frac{\beta_{j}^{\left(n\right)}}{T_{in}}(t_{vj}^{(n)-}-t_{vj}^{(n)+}))+b_{k}^{\left(p\right)}\\ \;=\sum_{j=0}^{N}w_{jk}^{\left(p\right)}\cdot \frac{\beta_{j}^{\left(n\right)}}{T_{in}}(t_{vj}^{(n)-}-t_{vj}^{(n)+}), \end{array}$$

where ReLU is used as the activation function, and the bias $b_{k}^{\left(p\right)}=w_{0k}^{\left(p\right)}$ is represented in the time-domain model by


(32) $$\begin{array}{l} b_{k}^{\left(p\right)}\;=w_{0k}^{\left(p\right)}\cdot 1\\ \;=w_{0k}^{\left(p\right)}\cdot \frac{\beta_{0}^{\left(n\right)}}{T_{in}}(t_{v0}^{(n)-}-t_{v0}^{(n)+}), \end{array}$$

in which $t_{v0}^{\left(n\right)+}=T_{in}$ is paired to $w_{0k}^{\left(p\right)}$, $t_{v0}^{\left(n\right)-}=0$ is paired to $-w_{0k}^{\left(p\right)}$ and $\beta_{0}^{\left(n\right)}=1$ as there is no input to the bias.

In the MLP shown in Figure 3, we transfer the output timings $t_{vj}^{\left(n\right)+}$ and $t_{vj}^{\left(n\right)-}$ generated in layer *n* to the neurons in layer *p* and perform the time-domain weighted-sum operation. The timings $t_{vk}^{\left(p\right)+}$ and $t_{vk}^{\left(p\right)-}$ are assumed to be produced at the *k*-th neuron of layer *p*. We relate timing $t_{vj}^{\left(n\right)+}$ to weight $w_{jk}^{\left(p\right)}$ and $t_{vj}^{\left(n\right)-}$ to $-w_{jk}^{\left(p\right)}$. We also assume here *N* = 3 and that $w_{1k}^{\left(p\right)}\geq 0,w_{2k}^{\left(p\right)}<0,w_{3k}^{\left(p\right)}\geq 0,b_{k}^{\left(p\right)}\geq 0$, and $\theta_{k}^{\left(p\right)+}=-\theta_{k}^{\left(p\right)-}$, where $\theta_{k}^{\left(p\right)+}$ and $\theta_{k}^{\left(p\right)-}$ are the threshold values for positively and negatively weighted-sum operations, respectively. Thus, according to Equation 4, we can obtain


(33) $$\begin{array}{l} w_{1k}^{\left(p\right)}(t_{vk}^{(p)+}-t_{v1}^{(n)+})+(-w_{2k}^{\left(p\right)})(t_{vk}^{(p)+}-t_{v2}^{(n)-})\\ \;+w_{3k}^{\left(p\right)}(t_{vk}^{(p)+}-t_{v3}^{(n)+})+b_{k}^{\left(p\right)}(t_{vk}^{(p)+}-t_{v0}^{(n)+})=\theta_{k}^{(p)+} \end{array}$$

(34) $$\begin{array}{l} \;(-w_{1k}^{\left(p\right)})(t_{vk}^{(p)-}-t_{v1}^{(n)-})+w_{2k}^{\left(p\right)}(t_{vk}^{(p)-}-t_{v2}^{(n)+})\\ +(-w_{3k}^{\left(p\right)})(t_{vk}^{(p)-}-t_{v3}^{(n)-})+(-b_{k}^{\left(p\right)})(t_{vk}^{(p)-}-t_{v0}^{(n)-})=\theta_{k}^{(p)-} \end{array}$$

By adding Equation 33 to Equation 34 on the left and right sides, respectively, the following relationship is obtained:


(35) $$\begin{array}{l} \overset{N=3}{\underset{j=0}{\sum }}||w_{jk}^{\left(p\right)}||\cdot \left(t_{vk}^{\left(p\right)+}-t_{vk}^{\left(p\right)-}\right)+\overset{N=3}{\underset{j=0}{\sum }}w_{jk}^{\left(p\right)}\cdot \left(t_{vj}^{\left(n\right)-}-t_{vj}^{\left(n\right)+}\right)=0. \end{array}$$

Thus, we can obtain the following simple expression:


(36) $$\begin{array}{l} \overset{N=3}{\underset{j=0}{\sum }}w_{jk}^{\left(p\right)}\cdot \left(t_{vj}^{\left(n\right)-}-t_{vj}^{\left(n\right)+}\right)=\left(t_{vk}^{\left(p\right)-}-t_{vk}^{\left(p\right)+}\right)\cdot \overset{N=3}{\underset{j=0}{\sum }}||w_{jk}^{\left(p\right)}||. \end{array}$$

Therefore, we generalize the number of neurons *N* = 3 to *N* again, and replace Equation 36 with Equation 31. Then, the output $y_{k}^{\left(p\right)}$ in Equation 31 can finally be


(37) $$\begin{array}{l} y_{k}^{\left(p\right)}=\sum_{j=1}^{N}w_{jk}^{\left(p\right)}\cdot ReLU(\sum_{i=0}^{N}w_{ij}^{\left(n\right)}\cdot x_{i})+b_{k}^{\left(p\right)}\\ \;=(t_{vk}^{(p)-}-t_{vk}^{(p)+})\cdot \sum_{j=0}^{N}\left\|w_{jk}^{\left(p\right)}\right\|\cdot \frac{\beta_{j}^{\left(n\right)}}{T_{in}} \end{array}$$

As a result, for neurons in the hidden layer *n*, we apply the time-domain weighted-sum operation to generate the timing $t_{vj}^{\left(n\right)+}$ and $t_{vj}^{\left(n\right)-}$ for the positively and negatively weighted-sum calculation from the input layer, respectively. Then, these timings are directly transferred to neurons in the next layer *p*, and timing $t_{vj}^{\left(p\right)+}$ and $t_{vj}^{\left(p\right)-}$ are obtained. Finally, we calculate the final outputs of the MLP using Equation 37 without calculating the middle layers' weighted-sum results using Equation 27.

**Figure 4:**
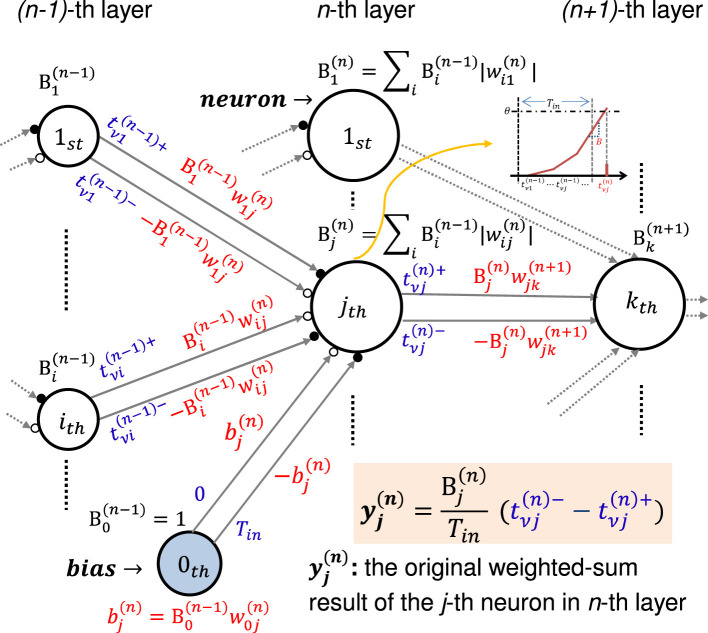
Summary of the general scheme of the time-domain weighted-sum neural network model.

We summarized the above mathematical time-domain operations in general MLPs graphically as shown in Figure 4. We refer to the “sum of the weights” as the “sum of the weights' absolute values” in the remainder of this paper. Note that the weights in the middle and output layers are replaced by the products of the original weight and the sum of the neuron's weights in the previous layer during the time-domain process. We indicated the sum of the new reconfigured weights by *B* instead of the aforementioned β, which indicated the sum of the original weights, as follows:


(38) $$\begin{array}{lll} \beta_{j}^{\left(1\right)} & = & \underset{i=0}{\sum }||w_{ij}^{\left(1\right)}||, \end{array}$$

(39) $$\begin{array}{l} B_{j}^{\left(2\right)}=\sum_{i=0}\beta_{j}^{\left(1\right)}\|w_{ij}^{\left(2\right)}\|\\ \;\cdots \end{array}$$

(40) $$\begin{array}{lll} B_{j}^{\left(n\right)} & = & \underset{i=0}{\sum }B_{j}^{\left(n-1\right)}||w_{ij}^{\left(n\right)}||. \end{array}$$

Note the bias of neuron *j* in layer *n*, whose index *i* = 0, is indicated as $B_{0}^{\left(n-1\right)}w_{0j}^{\left(n\right)}$, in which $B_{0}^{\left(n-1\right)}\equiv 1$. Therefore, the new weight connected from neuron *i* in layer *n*−1 to neuron *j* in layer *n* becomes $B_{i}^{\left(n-1\right)}w_{ij}^{\left(n\right)}$. Then we can have the original weighted-sum result of the *j*−th neuron in the *n*−th layer indicated by $y_{j}^{\left(n\right)}$ expressed as follows:


(41) $$\begin{array}{l} y_{j}^{\left(n\right)}=\frac{B_{j}^{\left(n\right)}}{T_{in}}\left(t_{\nu j}^{\left(n\right)-}-t_{\nu j}^{\left(n\right)+}\right) \end{array}$$

### 3.3 Numerical simulations of neural networks

**Figure 5:**
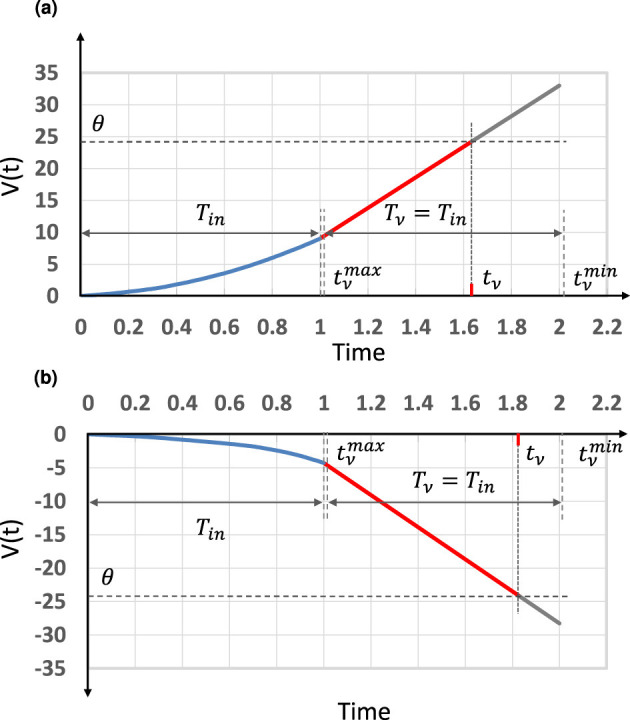
Simulation results for the time-domain weighted-sum calculation model applied to the neuron shown in Figure 2b: **(a)** PSP of positively weighted-sum operation with 249 inputs in which $T_{in}=1,\lambda =1,\epsilon =0.01,\beta^{+}=24.01$, and θ^+^ = 24.25. The output spike timing is $t_{\nu }^{+}=1.6356$. **(b)** PSP of negatively weighted-sum operation with 253 inputs in which $w_{0}=-0.06,w_{n+1}=-2.819,T_{in}=1,\lambda =1,,\epsilon =0.01,\beta^{-}=-24.01$, and θ^−^ = −24.25. The output spike timing is $t_{\nu }^{-}=1.8321$. Thus, the result of the weighted-sum calculation is $||\beta^{\pm }||\left(t_{\nu }^{-}-t_{\nu }^{+}\right)/T_{in}=4.718$.

We performed numerical simulations to verify our weighted-sum calculation model. First, in order to verify our model for weighted-sum calculation with different-signed weights, we conducted a simulation to perform a weighted-sum calculation with 501 pairs of inputs and weights that consisted of 249 positive and 252 negative weights. We added a dummy weight to make the sum of positive weights equal to the absolute sum of the negative ones. Figure 5 shows the simulation results of the time-domain weighted-sum calculation with a dummy weight *w*_*n*+1_. The results show that the weighted summation can be calculated correctly with different negative and positive firing timing inputs, each set of which are multiplied by the corresponding signed weights.

Then, we applied our model to a four-layer MLP (784-100-100-100-10) to classify the MNIST digit character set. We trained the MLP and then performed inference according to Equation 41 with the obtained weights, which were either binary (Courbariaux et al., 2015) or floating-point values. As described above, output spike timings at each neuron in the previous layer were directly conveyed to the neurons in the next layer without obtaining weighted-sum results in the middle layers. We found that we obtained the same weighted-sum calculation results in the last layer and also the same recognition precisions in both NNs as in the numerically calculated ones.

## 4 Issues about time-domain weighted-sum models toward VLSI implementation

We have established our time-domain weighted-sum neural network model in a general form and summarized it in Figure 4, and conducted numerical simulations that verified the effectiveness in pre-trained ANN models in Section 3. In this section, we will discuss some issues about the model when implemented in analog VLSI circuits.

### 4.1 Weights and biases

**Figure 6:**
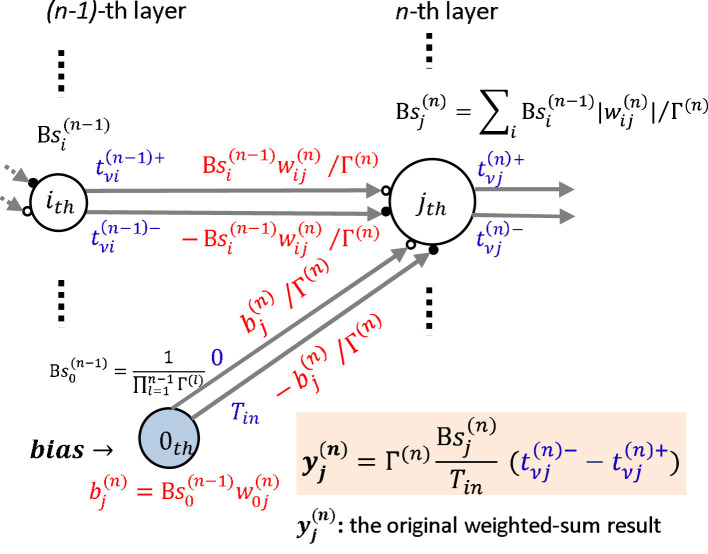
Weights scaling: a scaling factor Γ^(*n*)^, expressed in Equation 52, is introduced for the *n*-th layer to adjust the reconfigured large PSP slope shown in Figure 4 to a reasonable level. After scaling, the slopes are expressed as $Bs_{i}^{\left(n-1\right)}w_{ij}^{\left(n\right)}/\Gamma^{\left(n\right)}$, where $Bs_{i}^{\left(n-1\right)}$ represents the scaled sum of weights in the previous layer *n*−1 and is scaled by Γ^(*n*−1)^.

In the general time-domain weighted-sum neural network model as shown in Figure 4 and Equation 41, the weights must be reconfigured as $B_{i}^{\left(n-1\right)}w_{ij}^{\left(n\right)}$ in order to generate two timings whose interval is proportional to the original weighted-sum result, which results in the same recognition accuracy as the original ANN. The reconfigured weights correspond to the PSP slope in the IF neuron model. The slope will be greatly increased with the reconfiguration, which may result in very high potentials that do not satisfy the hardware system criteria. To solve this problem, we introduced a scaling factor Γ^(*n*)^ for the *n*-th layer as shown in Figure 6, to adjust the PSP slope to a reasonable level. Note that every neuron in the same layer has the same scaling factor.

Then the reconfigured weight becomes $Bs_{i}^{\left(n-1\right)}w_{ij}^{\left(n\right)}/\Gamma^{\left(n\right)}$, where $Bs_{i}^{\left(n-1\right)}$ represents the scaled sum of weights in the previous layer *n*−1, and the scaled sum of the reconfigured weights in layer *n*, which is also interpreted as the total PSP slope, is expressed as


(42) $$\begin{array}{lll} Bs_{j}^{\left(1\right)} & = & \frac{1}{\Gamma^{\left(1\right)}}\underset{i=0}{\sum }||w_{ij}^{\left(1\right)}||, \end{array}$$

(43) $$\begin{array}{l} Bs_{j}^{\left(2\right)}=\frac{1}{\Gamma^{\left(2\right)}}\sum_{i=0}Bs_{j}^{\left(1\right)}\|w_{ij}^{\left(2\right)}\|\\ \;\cdots \end{array}$$

(44) $$\begin{array}{lll} Bs_{j}^{\left(n\right)} & = & \frac{1}{\Gamma^{\left(n\right)}}\underset{i=0}{\sum }Bs_{j}^{\left(n-1\right)}||w_{ij}^{\left(n\right)}|| \end{array}$$

so we can obtain


(45) $$\begin{array}{lll} Bs_{j}^{\left(1\right)} & = & \frac{1}{\Gamma^{\left(1\right)}}\beta_{j}^{\left(1\right)}, \end{array}$$

(46) $$\begin{array}{l} Bs_{j}^{\left(2\right)}=\frac{1}{\prod_{l=1}^{\left(2\right)}\Gamma^{\left(l\right)}}B_{j}^{\left(2\right)}\\ \;\cdots \end{array}$$

(47) $$\begin{array}{lll} Bs_{j}^{\left(n\right)} & = & \frac{1}{\prod_{l=1}^{\left(n\right)}\Gamma^{\left(l\right)}}B_{j}^{\left(n\right)} \end{array}$$

Note that the bias $b_{j}^{\left(n\right)}$ is reconfigured as


(48) $$\begin{array}{l} b_{j}^{\left(n\right)}=Bs_{0}^{\left(n-1\right)}w_{0j}^{\left(n\right)}/\Gamma^{\left(n\right)}, \end{array}$$

where $Bs_{0}^{\left(n-1\right)}=\frac{1}{\prod_{l=1}^{\left(n-1\right)}\Gamma^{\left(l\right)}}$. Accordingly, the original weighted-sum result is expressed as


(49) $$y_{j}^{\left(n\right)}=\prod_{l=1}^{\left(n\right)}\Gamma^{\left(l\right)}\frac{Bs_{j}^{\left(n\right)}}{T_{in}}(t_{\nu j}^{(n)-}-t_{\nu j}^{(n)+})=\frac{B_{j}^{\left(n\right)}}{T_{in}}(t_{\nu j}^{(n)-}-t_{\nu j}^{(n)+})$$

From Equations 49, 41, we can find that the difference between timing $t_{\nu j}^{\left(n\right)-}$ and $t_{\nu j}^{\left(n\right)+}$ remains the same before and after the scaling operations.

**Figure 7:**
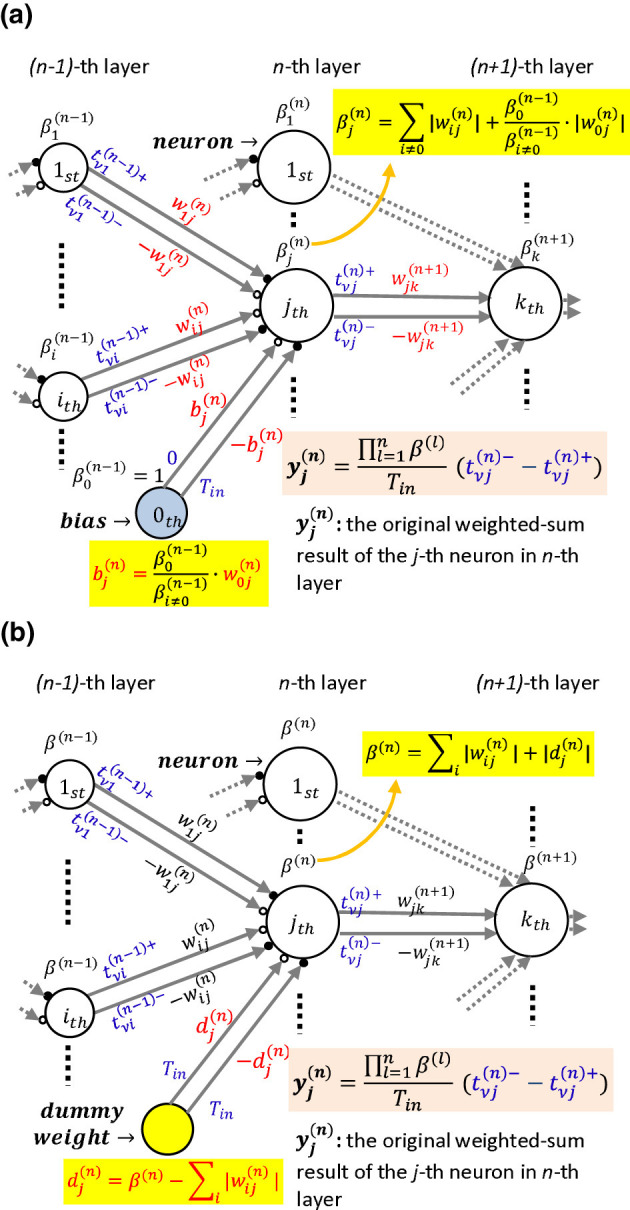
Derivations from the general time-domain weighted-sum process in MLPs whose weights (biases not included) involved in the time-domain process, i.e., $B_{j}^{\left(n-1\right)}w_{ij}^{\left(n\right)},i\neq 0$, are scaled to the original weights, $w_{ij}^{\left(n\right)},i\neq 0$: **(a)** the models with biases: the biases are scaled as $\frac{\beta_{0}^{\left(n-1\right)}}{\beta_{i\neq 0}^{\left(n-1\right)}}\cdot w_{0j}^{\left(n\right)}$ under the assumption of Equations 50, 51 accompanying the weights' scaling operation; **(b)** the models without biases: we add an extra dummy weight $d_{j}^{\left(n\right)}=\beta^{\left(n\right)}-\underset{i}{\sum }||w_{ij}^{\left(n\right)}||$ whose two input timings are the same, i.e., the input is 0, for neuron *j* in layer *n* to make the sum of weights of every neuron in layer *n* identical, which is marked as β^(*n*)^.

So far we have shown the general scaling process toward the weights' reconfiguration. Next, we will show some special cases that can simplify the weights' reconfiguration. Suppose that


(50) $$\begin{array}{lll} \underset{i\neq 0}{\sum }||w_{i1}^{\left(n\right)}|| & = & \underset{i\neq 0}{\sum }||w_{i2}^{\left(n\right)}||=\cdots =\underset{i\neq 0}{\sum }||w_{ij}^{\left(n\right)}||, \end{array}$$

(51) $$\begin{array}{lll} ||w_{01}^{\left(n\right)}|| & = & ||w_{02}^{\left(n\right)}||=\cdots =||w_{0j}^{\left(n\right)}|| \end{array}$$

meaning that the bias and the sum of the original weights of every neuron in layer *n* are equal to each other, such as in the BinaryConnect NN model (Courbariaux et al., 2015), whose weights and biases are binary values. Let the scaling factor Γ^(*n*)^ be


(52) $$\begin{array}{l} \Gamma^{\left(1\right)}=\beta_{j}^{\left(0\right)},\\ \Gamma^{\left(2\right)}=\beta_{j}^{\left(1\right)},\\ \;\cdots \\ \Gamma^{\left(n\right)}=\beta_{j}^{\left(n-1\right)} \end{array}$$

where $\beta_{j}^{\left(l\right)}$ denotes the sum of the weights of the layer *l*(= 0, 1, 2⋯*n*) expressed as follows:


(53) $$\begin{array}{l} \beta_{j}^{\left(0\right)}=1,\\ \beta_{j}^{\left(1\right)}=\sum_{i\neq 0}\left\|w_{ij}^{\left(1\right)}\right\|+\frac{1}{\beta_{j\neq 0}^{\left(0\right)}}\|w_{0j}^{\left(1\right)}\|,\\ \beta_{j}^{\left(2\right)}=\sum_{i\neq 0}\left\|w_{ij}^{\left(2\right)}\right\|+\frac{1}{\beta_{j\neq 0}^{\left(1\right)}}\|w_{0j}^{\left(2\right)}\|,\\ \;\cdots \\ \beta_{j}^{\left(n\right)}=\sum_{i\neq 0}\left\|w_{ij}^{\left(n\right)}\right\|+\frac{1}{\beta_{j\neq 0}^{\left(n-1\right)}}\|w_{0j}^{\left(n\right)}\|, \end{array}$$

where $\beta_{j=0}^{\left(n\right)}=1$. Note that $\beta_{1}^{\left(n\right)}=\beta_{2}^{\left(n\right)}=\cdots =\beta_{j}^{\left(n\right)}$ under the assumption of Equations 51, 52. Then we can generate the desired timings using only the original weights $w_{ij}^{\left(n\right)},i\neq 0$ shown in Figure 7a, without reconfiguring the weights (not biases included) as $B_{i}^{\left(n-1\right)}w_{ij}^{\left(n\right)},i\neq 0$ shown in Figure 4. However, the bias $b_{j}^{\left(n\right)}$must be reconfigured as


(54) $$\begin{array}{l} b_{j}^{\left(n\right)}=\frac{1}{\beta_{j}^{\left(n-1\right)}}\cdot w_{0j}^{\left(n\right)} \end{array}$$

Accordingly, the original weighted-sum result will be


(55) $$\begin{array}{l} y_{j}^{\left(n\right)}=\frac{\prod_{l=1}^{\left(n\right)}\beta^{\left(l\right)}}{T_{in}}\left(t_{\nu j}^{\left(n\right)-}-t_{\nu j}^{\left(n\right)+}\right) \end{array}$$

where β^(*l*)^ denotes the identical value of the sum of weights among neurons in layer *l*.

In modern deep neural networks with deep layers and a large number of parameters, several experiments using models without bias demonstrated that there was an accuracy degradation of 3.9% and 4% in CIFAR10 and CIFAR100 datasets, respectively (Wang et al., 2019). Such degradation of less than 5% is supposed to be acceptable when deploying DNNs to resource-constrained edge devices, in which trade-offs between accuracy, latency, and energy efficiency need to be carefully considered (Shuvo et al., 2022; Ngo et al., 2025).

**Figure 8:**
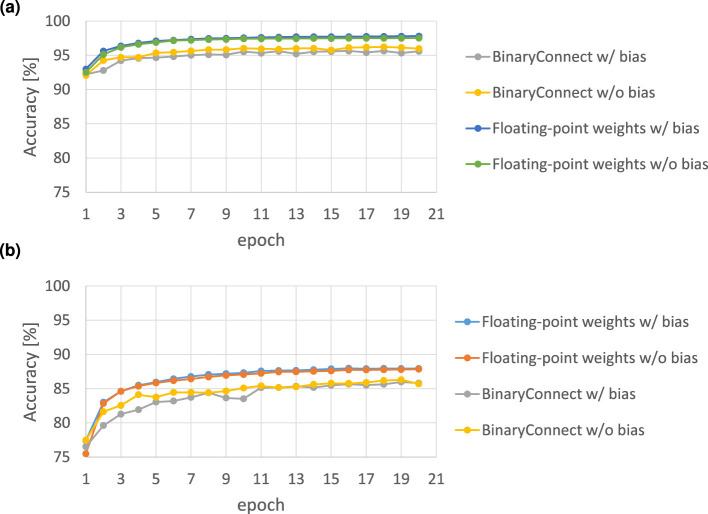
The MLP recognition accuracy comparison between models with and without bias on **(a)** MNIST dataset and **(b)** Fashion-MNIST dateset. The MLP models are the BinaryConnect model and the general floating-point connection model.

We also trained a four-layer MLP (784-100-100-100-10) with and without biases on the datasets MNIST and Fashion-MNIST and compared the results in both the floating-point and binary weight connect models, as shown in Figure 8. The results showed that the accuracies with and without biases were comparable. Therefore, in certain cases, the bias can be removed so that the reconfiguration cost of the bias shown in Equations 48, 54 is saved.

We have shown a case in which the weights and biases were restricted to the condition shown in Equations 50, 51 so that the cost of the weights' reconfiguration can be saved. Next, we propose a method to satisfy the restriction in Equation 50 for a more general ANN model to save the weights' reconfiguration shown in Figure 7b. Note that we discuss the method in the model without biases for simplicity. We add a dummy weight to every neuron in layer *n* to make the sum of the weights identical. We regard the identical value in layer *n* as β^(*n*)^. The dummy weight to neuron *j* in layer *n*, indicated as $d_{j}^{\left(n\right)}$, is allocated as:


(56) $$\begin{array}{l} d_{j}^{\left(n\right)}=\beta^{\left(n\right)}-\underset{i}{\sum }||w_{ij}^{\left(n\right)}|| \end{array}$$

Note that the input timings for the dummy weights $d_{j}^{\left(n\right)}$ and $-d_{j}^{\left(n\right)}$ are identical, such as *T*_*in*_, meaning a 0 input.

### 4.2 Output timing difference

From Equations 40, 41, we can find that the coefficients applied to the output timing difference are increased monotonically as the layer goes deeper. It has generally been observed that the outputs of every neuron, i.e., the weighted-sum results, converge at a certain range in a well-trained ANN. Therefore, we figure out that the timing difference decreases monotonically as the layer goes deeper. This effect essentially results from our calculating the positively signed and negatively signed weighted sums separately.

**Figure 9:**
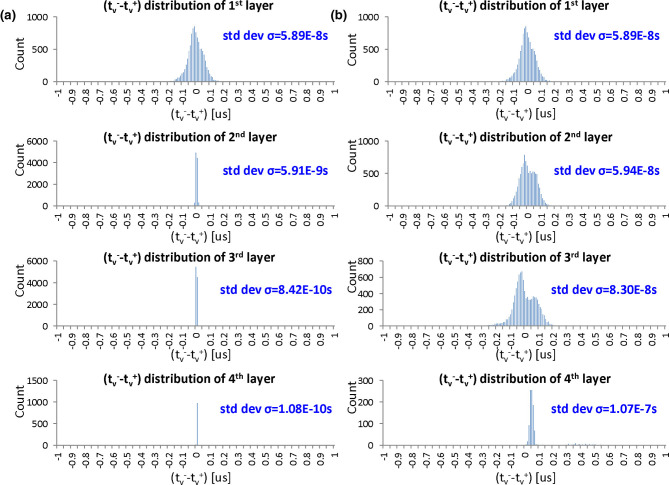
The distribution of the output timing difference, $t_{\nu }^{-}-t_{\nu }^{+}$, in every layer of the 784-100-100-100-10 four-layer MLP model *T*_*in*_ is assumed to be 1 μs: **(a)** the original distributions; **(b)** the distributions in which the timing differences are amplified with a gain of 10.

**Figure 10:**
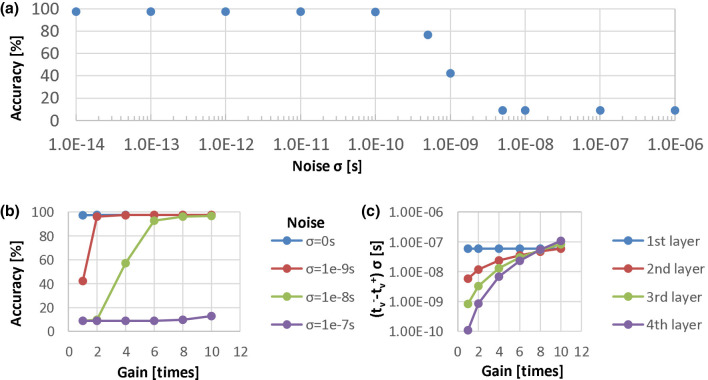
Noise tolerance examinations in the four-layer MLP: **(a)** recognition accuracy of the model without timing difference amplification evaluated under different noise levels; **(b)** recognition accuracy under the different amplification gain conditions with the critical noise injected; **(c)** the output timing difference distributions in every layer under different amplifying gain conditions.

We demonstrate the timing difference issue by means of a case study in which we perform the time domain weighted-sum inference in a well-trained four-layer MLP (784-100-100-100-10). We collected the distributions of the output timing differences in every layer of the MLP when performing evaluation on the 10,000-sample test data in MNIST. Figure 9a shows the distributions where the histograms show only 100 of the total 10,000 samples, but the standard deviations σ are calculated over the total samples. The output timing differences, σ, of the 1st, 2nd, 3rd and 4th layers are 5.89e − 8 s, 5.91e − 9 s, 8.42e − 10 s, and 1.08e − 10 s, respectively, under the assumption of *T*_*in*_ = 1 μs. If we assume that the resolution time step is 10 ns by taking the noise of analog circuits into account, the great majority of the timing differences in the 2nd and the subsequent layers are less than the time resolution. Therefore, such a time-domain multi-layer model cannot be implemented into analog VLSI directly. We also conducted an experiment to evaluate the noise tolerance of the above model. In the experiment, we injected noise with different standard deviations σ to the output timing $t_{\nu }^{-}$ and $t_{\nu }^{+}$, and evaluated the MLP recognition accuracy. The results are shown in Figure 10a. We can find that the accuracy deteriorates when the noise level is around 0.5 ns near the distribution σ of the 3rd layer. The model does not work when σ is over 5 ns near that of the 2nd layer.

In order to solve the problem of decreasing output timing difference, we can introduce an amplification component into our model, which can amplify the timing difference just before the timings are transferred to the next layer. To examine the effectiveness of this amplification function, we performed experiments in which we amplified the timing difference with different gains and evaluated the recognition accuracy with the critical noise injected. The results are shown in Figure 10b. We also plotted the output timing difference distributions in every layer under different amplifying gain conditions. Figure 10c shows the distribution standard deviations, and Figure 9b shows the histograms with a gain of 10. Note that in these experiments, we simply set the same amplification gains in every layer without optimizing the gains. We found that the recognition accuracy is comparable to that in the model without noise injected if we select a gain to make the output timing difference distribution σ of the last layer larger than the critical noise level, such as 10 ns. However, for more robustness, the distribution σ is supposed to be much larger than the resolution time step so that the gain can be 8–10. We verified the effectiveness of the amplification function and established the time-domain weighted-sum model with amplification components. In VLSI circuits, we can introduce a time-difference-amplifier (TDA) (Abas et al., 2002; Asada et al., 2018) component to amplify the output timing difference.

## 5 Circuits and architectures for TACT-based neural networks

**Figure 11:**
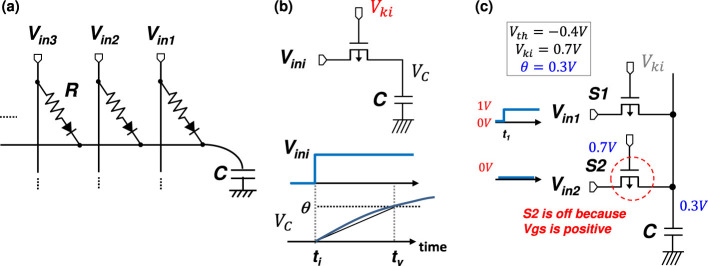
Synapse circuit: **(a)** step voltage input and a resistance-capacitance (R-C) circuit in which a pMOSFET acts as resistance *R*, and parasitic capacitance of interconnection and the gate capacitance of MOSFETs act as *C* in a VLSI circuit; **(b)** approximately linear response of the step voltage input at timing *t*_*i*_ with a slope determined by gate voltage *V*_*ki*_. **(c)** an operating example for explanation of the pMOSFET synapse's rectification function.

As a VLSI implementation of our time-domain weighted-sum calculation based on the TACT approach, we propose an RC circuit in which a capacitor is connected by multiple resistors, as shown in Figure 11a. Theoretical estimations have indicated that this circuit can perform weighted-sum calculations with extremely low energy consumption (Tohara et al., 2016; Wang et al., 2016).

In CMOS VLSI implementation, resistance *R* can be replaced by a p-type MOS field-effect transistor (pMOSFET), as shown in Figure 11b. The approximately linear slope *k* is generated by capacitance *C* and ON resistance of a pMOSFET with a step voltage input *V*_*in*_, where we use step voltages instead of spike pulses as inputs. Each resistance should have a rectification function to prevent an inverse current. The rectification function is automatically realized by the FET operation as follows. When a pMOSFET receives a step-voltage input, the terminal voltage of the input is higher than that at *C*, and therefore, the input-side terminal of the pMOSFET is the “source,” and the capacitor-side terminal is the “drain.” In this state, if the gate-source voltage (*V*_*gs*_) of the pMOSFET is set to exceed its threshold voltage, the pMOSFET turns on, and *C* is charged up. On the other hand, when a pMOSFET receives no input, the terminal voltage of the input is lower than that at *C*, and therefore the source-drain position in the pMOSFET is reversed; i.e., the input-side terminal of the pMOSFET is “drain,” and the capacitor-side terminal is “source.” In this state, if the *V*_*gs*_ of the pMOSFET is set not to exceed its threshold voltage, the pMOSFET turns off, and the charges stored at *C* do not flow back to the input side. An operating example is shown in Figure 11c, in which the synapse pMOSFET without input (i.e., the input voltage is 0V here), denoted as S2, is strongly off because its gate-source voltage is positive.

### 5.1 Architectures

**Figure 12:**
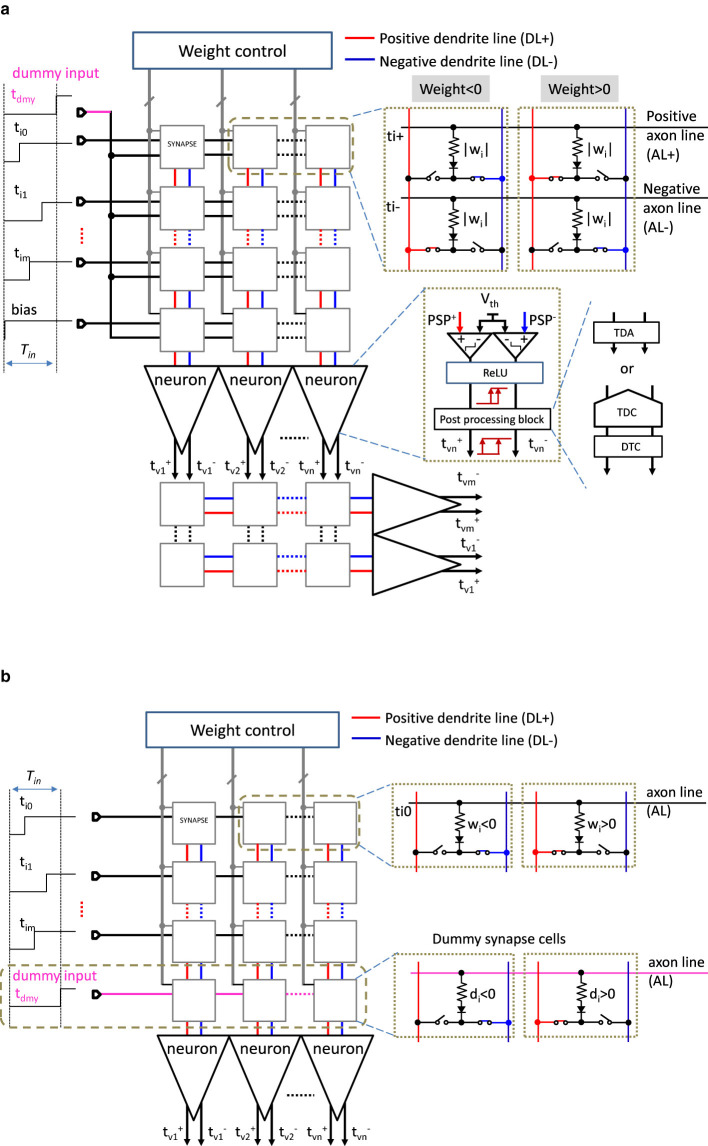
TACT-based MLP architecture. **(a)** A two-layer MLP architecture described in Figure 3 whose input layer is modeled in Figure 2c in which each synapse has two set of inputs and weights. **(b)** Another type of input layer architecture of the MLP that there is one input and one weight for each synapse while a row of dummy cells with a dummy input are added.

We propose a circuit architecture of a neural network based on our established weighted-sum calculation model which is suitable for our TACT approach, accommodating both positive and negative weights. The architecture is shown in Figure 12 and is composed of a crossbar synapse array acting as resistive elements, the neuron part functioning as thresholding and nonlinear activation, and the configuration part controlling synapses.

Figure 12a shows a two-layer MLP architecture described in Figure 3 whose input layer is modeled in Figure 2c in which each synapse has two sets of inputs and weights. Another type of input layer architecture of the MLP is shown in Figure 12b, as described in Figure 2b.

In Figure 12a, there are two inputs for each synapse circuit, which are *t*_*i*_ as the signal input and *t*_*dmy*_ as a dummy input in the first layer, and $t_{\nu i}^{+}$ and $t_{\nu i}^{-}$ in the subsequent layers. Pairs of positive and negative timings are directly connected to the next layer without subtracting the negatively signed weighted results from the positively signed weighted results according to the theory explained in Section 3.2 and Figure 4. In the synapse array, the horizontal and vertical lines are referred to as “axons” of the previous neurons and “dendrites” of the post neurons, respectively. Suppose that each axon line has *M* synapse circuits, and each dendrite line receives *N* synapse outputs. A synapse cell is designed with two resistive elements and two pairs of switches. A set of two identical resistances represents the weight value. The resistive elements are expected to be replaced by resistance-based analog memories to store the multi-bit weights (Sebastian et al., 2020). We can assume that the upper-side axon is for $t_{\nu i}^{+}$ and the other is for $t_{\nu i}^{-}$, and the left-side dendrite is for a positive weight connection while the other is for a negative one. The two switches are exclusively controlled according to the corresponding sign of weights, which is controlled by the weight control circuit. By contrast, there is one input and one weight for each synapse in the input layer shown in Figure 12b, while a row of dummy cells with a dummy input is added to the synapse array conceptually according to Section 3.1. The resistances (*d*_*i*_) of the dummy cell are theoretically set according to Equation 56.

In AIMC, the most common implementation of a signed weight *w*_*i*_ is using a differential scheme with two subweights $w_{i}^{+}$ and $w_{i}^{-}$ such that


(57) $$\begin{array}{l} w_{i}=w_{i}^{+}-w_{i}^{-}, \end{array}$$

in which one is for positive weighted-sum and another is for negative one (Xiao et al., 2023; Aguirre et al., 2024). Here, we treat the following signed weight configuration as a special differential scheme (Yamaguchi et al., 2020; Kingra et al., 2022):


(58) $$w_{i}=\{\begin{array}{ll} w_{i}^{+}-0 & \text{where }w_{i}^{-}\text{ is disabled, indicating }w_{i}\geq 0,\\ 0-w_{i}^{-} & \text{where }w_{i}^{+}\text{ is disabled, indicating }w_{i}\leq 0 \end{array}.$$

Subtraction to obtain the final weighted-sum result is commonly performed in either differential mode or common mode. In differential mode, the operation is carried out at two separate nodes within the peripheral circuitry (Guo et al., 2017; Joshi et al., 2020; Yamaguchi et al., 2020; Sahay et al., 2020). In common mode, the subtraction is performed at a single node based on Kirchhoff's law, using either bipolar (Wan et al., 2022; Aguirre et al., 2024) or unipolar inputs (Wang et al., 2021; Khaddam-Aljameh et al., 2022; Le Gallo et al., 2023). It's worth noting that common-mode subtraction with unipolar inputs generally requires a bi-directional peripheral circuit, capable of providing two voltages: one higher and one lower than the common node voltage.

Signed inputs for 4-quadrant computation can be implemented by applying opposite polarity voltage (Marinella et al., 2018; Le Gallo et al., 2024) or using differential pairs when the input is unipolar (Schlottmann and Hasler, 2011; Bavandpour et al., 2019a). Additionally, signed computations without adopting the above two designs need multiple phase modulations, like two phases in Kingra et al. (2022) for 2-quadrant MAC and four phases in Le Gallo et al. (2023) for 4-quadrant MAC.

With respect to the signed weight representation in our approaches, the configuration in Figure 12b is regarded as the special differential scheme described in the expression Equation 58, and that in Figure 12a restricts the two subweights to be identical. We term the latter configuration as a *complementary scheme*, distinguishing it from the general differential scheme. With respect to the signed input representation, we adopt differential pairs as in Bavandpour et al. (2019a). Our model with the complementary scheme can perform four-quadrant MAC computation in a single modulation without bipolar or bi-directional peripheral requirements.

**Figure 13:**
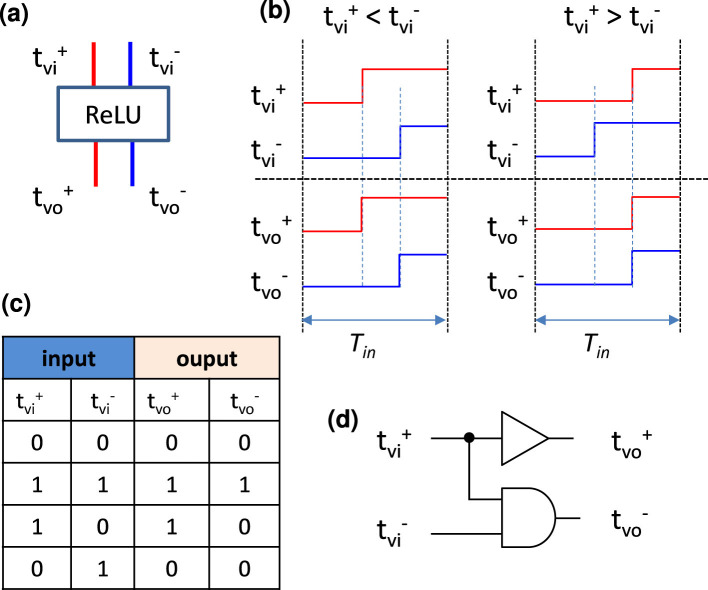
ReLU block: **(a)** symbol of the block; **(b)** timing chart of the ReLU function; **(c)** true table and **(d)** circuit implementation by simple logic gates.

The neuron part shown in Figure 12a consists of a thresholding block, such as a comparator, a ReLU block, and a post-processing block (PPB) after the ReLU block. ReLU block with input and output timings is shown in Figure 13a. The relationships between inputs and outputs are illustrated in Figure 13b, in which both output timings are set identical to $t_{\nu i}^{+}$ when $t_{\nu i}^{+}>t_{\nu i}^{-}$. The truth table is shown in Figure 13c and accordingly the ReLU activation function can easily be implemented by logic gates, as shown in Figure 13d. With such circuits, the nonlinear activation function ReLU can be implemented with low energy consumption operation. The PPB can be either a TDA circuit or a set of TDC and DTC to address the issue of timing difference shrinkage discussed in Section 4.2. The TDA is introduced to transmit the timings to the next layer directly in an analog manner, and the TDC and DTC are introduced to communicate intra-layers digitally. We leave the PPB implementation with high performance, such as high precision and low power, to be an open design problem in this paper.

We summarize the main differences of our complementary weight approach with respect to the previous similar research (Bavandpour et al., 2019a; Sahay et al., 2020), which are also partially inspired by our model (Morie et al., 2016; Tohara et al., 2016; Wang et al., 2018), as follows:

- Input and output information are encoded as the timing of step voltages, rather than using a PWM scheme.
- The response to every input step voltage in the output line is continuous until the firing threshold of the post-neuron, instead of being discrete.
- We represent signed weights using a complementary scheme, where the two sub-weights are identical, rather than using a differential scheme.

### 5.2 Circuits

In order to evaluate the energy consumption and computation precision of our TACT-based circuit, we designed a PoC CMOS circuit equivalent to the RC circuit to perform the one-column (i.e., *N* inputs and 1 output) signed weighted-sum calculation whose synapses are in a complementary scheme. The resistive elements in the synapses are replaced by pMOSFETs. The comparator function in the neuron part is implemented by an S-R latch.

**Figure 14:**
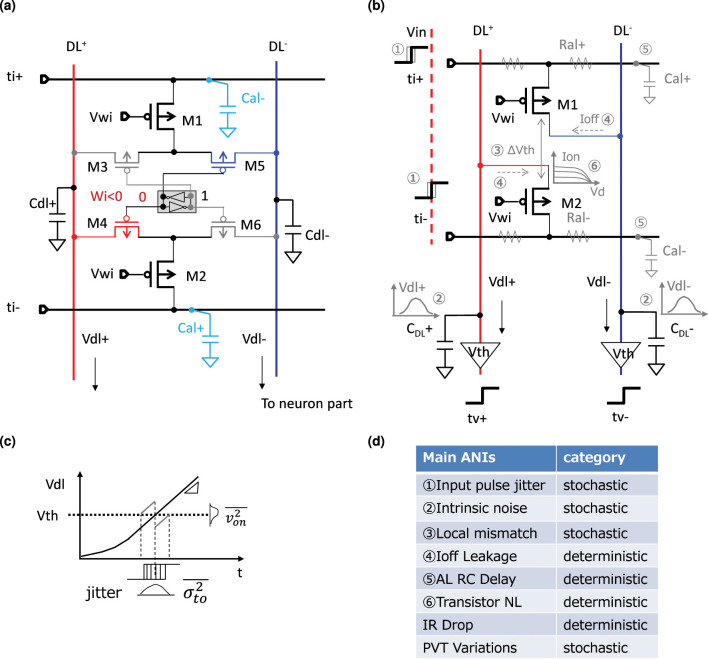
SRAM based synpase circuit and the main analog nonidealities (ANIs): **(a)** SRAM based synpase circuit; **(b)** Main ANIs of the synapse circuit **(c)** Output timing jitters induced by the ANIs **(d)** Main ANIs summary.

We propose SRAM-based synapse circuits to implement an IMC circuit for the computation shown in Figure 14a. It consists of a 1-bit standard 6T SRAM to save the sign of a weight, a pair of pMOSFETs assigned as M1 and M2 serving as the value of the weight, and four pMOSFETs assigned as M3- M6 functioning as switches controlled by the SRAM state. M1 and M2 are identical transistors and are biased with the same gate voltage, implementing the concept of complementary dummy weight introduced in the previous chapter. They serve as current sources operating in the subthreshold saturation region, showing a high impedance. M3-M6 switch the current to the dendrite line determined by the weight's sign according to the diagram shown in Figure 12a. As a PoC circuit, we implemented the BinaryConnect NN (Courbariaux et al., 2015) by limiting all the biases of the synapse pMOSFETs to be the same.

The main design parameters and simulation conditions are summarized in Table 1. We used the predictive technology model (PTM) 45 nm SPICE model for the design and simulation. Both the gate length and width of the synapse pMOSFET were 0.45 μm. Based on the size of the synapse pMOSFET, we estimated the parasitic capacitance of the axon line and the dendrite line based on 65 nm SRAM-based IMC circuits (Kneip and Bol, 2021). As a result, the parasitic capacitance of the axon line per cell, denoted as *C*_*al*_, is around 0.88 fF, and the parasitic capacitance of the dendrite line per cell, denoted as *C*_*dl*_, is around 0.87 fF. *V*_*gs*_ of the synapse pMOSFET is fixed at –0.34 V so that one synapse current *I*_*s*_ is around 11.5 nA under typical conditions. We set the typical supply voltage of the synapse array and the neuron part to be 1.1 and 0.75 V, respectively. And the threshold (*V*_*TH*_) of the post-neuron (i.e., the S-R latch) is around 0.4 V typically.

**Table 1:** Simulation conditions.

**Item**	**Unit**	**Values**		
Technology	–	PTM 45 nm		
Dimension of the synapse pMOSFET	μm	*L* = 0.45 *W* = 0.45		
Process corner	–	tt	ss	ff
Vth of pMOSFET	V	–0.423	–0.452	–0.392
Temperature	°C	27	–30	90
Vgs of the pMOSFET	V	fixed to –0.34		
Drain current of the pMOSFET	nA	11.5	4.3	27.8

With respect to the computation precision, we set the full-scale time window (*T*_*in*_) to be 640 ns, and the effective number of bit (ENOB) to be 4 bit as the design target. Then the total capacitance of the dendrite line (*C*_*DL*_) for MAC computation can be obtained by


(59) $$\begin{array}{c} C_{DL}=\frac{NI_{s}T_{in}}{V_{TH}}. \end{array}$$

*C*_*DL*_ includes the total parasitic capacitance of the dendrite lines, *NC*_*dl*_, the input capacitance of the post neuron, *C*_*i*_, and an extra load capacitor (*C*_*l*_) which is needed under the given *T*_*in*_ and *I*_*s*_ conditions.

Analog computation suffers from analog nonidealities (ANIs) (Kneip and Bol, 2021). These ANIs limit the computation precision, leading to a degradation of the inference accuracy. We sketch the main ANIs on our synapse circuit shown in Figure 14b. All these ANIs may cause the output jitters illustrated in Figure 14c resulting in computation errors. We summarize them in Figure 14d classifying them by their stochastic or deterministic nature.

Because time domain computation is sensitive to PVT variations (Seo et al., 2022) and local device mismatch is dominant than the intrinsic noise (Kneip and Bol, 2021; Gonugondla et al., 2021), we mainly considered the local mismatch and the PVT variations here.

**Figure 15:**
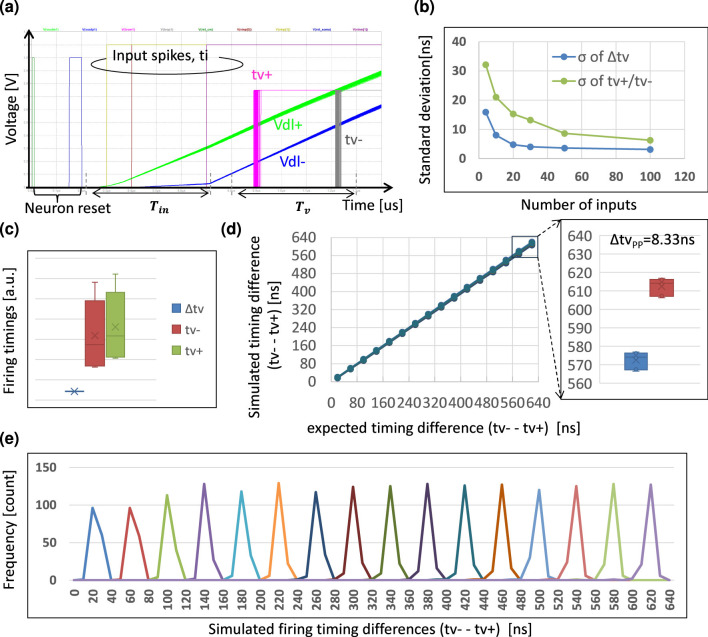
TACT-based time-domain weighted-sum simulation results: **(a)** operational waveforms in a Monte Carlo simulation; **(b)** Monte Carlo simulation results of the positive or negative dendrite line firing timings $t_{\upsilon }^{+}/t_{\upsilon }^{-}$ and their difference $\Delta t_{\upsilon }=t_{\upsilon }^{+}-t_{\upsilon }^{-}$ vs. the number of MAC's inputs (*N*_*in*_); **(c)** comparison of the simulated PVT variations between $t_{\upsilon }^{+}/t_{\upsilon }^{-}$ and Δ*t*_υ_; **(d)** simulated weighted-sum linearily and its PVT variation in which the max peak-to-peak (Δ*t*_υ*pp*_) is 8.33 ns in the case of *N*_*in*_ = 50; **(e)** the distribution of the 50-inputs weighted-sum results (Δ*t*_υ_) which is well divided into 16 levels based on the predefined 4-bit output time resolution.

We conducted Monte Carlo simulations to evaluate the errors induced by the local mismatches. A set of Monte Carlo simulation waveforms is shown in Figure 15a, and the standard deviations (σ) vs. the number of the inputs, *N*, are shown in Figure 15b. The results showed that the σ scales roughly as $\frac{1}{\sqrt{N}}$ leading to higher precision for larger *N* (Bavandpour et al., 2019a).

Our approach is expected to have good immunity to the PVT variations. The error induced by PVT variation in both the synapse array and neuron part is common to both positive and negative dendrite lines, and thus can be canceled from the point of view of the timing difference. To verify the PVT variation tolerance, we conducted a simulation for *N* = 50 with process and temperature (PT) corners shown in Table 1, and changed the supply voltage of the neuron part to 0.65, 0.75, and 0.85 V. We supposed that the gate voltage of the synapse pMOSFET changed along with the supply voltage of the synapse part, and thus the *V*_*gs*_ of it is fixed. One MAC PVT simulation result is shown in Figure 15c. The synapse current *I*_*s*_ changed largely across the PT variations shown in Table 1, resulting in large variation of the single dendrite line output timings $t_{\nu }^{+}/t_{\nu }^{-}$. However, the variation of the difference between them was much smaller. We also checked the weighted-sum linearity against the ideal 16 levels with 1*LSB* = 40 ns under the PVT simulations. The simulated linearity result is shown in Figure 15d, indicating good linearity with the max peak-to-peak variation of 8.33 ns. Finally, we incorporated the PVT and Monte Carlo simulation results into a distribution shown in Figure 15e, indicating that the ENOB = 4 was well achieved. We summarized the potentially achievable MAC computation precision with the number of inputs increased to 256, as shown in Table 2.

**Table 2:** The MAC computation precision.

**Item**	**Unit**	**Simulation results**	**Estimation results**	
Number of inputs	–	50	100	256
Full-scale time window (Tin)	ns	640		
Peak-to-peak of $t_{\nu }^{-}-t_{\nu }^{+}$ caused by PVT	ns	8.33	5.89	1.4
Standard deviation (σ) of $t_{\nu }^{-}-t_{\nu }^{+}$
caused by mismatch	ns	3.61	^*^3.12	1.99
ENOB computed by 1 σ	bit	5.3	5.7	6.3
ENOB computed by 3 σ	bit	4.4	4.7	5.4

^*^Simulation value.

With respect to energy consumption, an input voltage charges up the parasitic capacitance of the axon line, *C*_*al*_, and then charges the capacitance *C*_*DL*_ via synapse pMOSFET. Therefore, total energy consumption due to the dendrite line (DL) charge and discharge, *E*_*DL*_, is expressed by $E_{DL}=C_{DL}V_{TH}^{2}$, and it was 80.59 fJ when *V*_*TH*_ = 0.3 V and 143.27 fJ when *V*_*TH*_ = 0.4 V. And the total energy consumption per DL due to the axon line (AL) charge and discharge, *E*_*AL*_, is expressed by $E_{AL}=NC_{al}V_{dd}^{2}$, and it was 53.24 fJ, where *V*_*dd*_ = 1.1 V.

As for the neuron part, which consists of an S-R latch and the output buffer, the energy consumption, *E*_*NP*_, was about 216.91 fJ when the supply voltage was 0.75 V, and decreased to about 76.49 fJ when the supply voltage is 0.65 V.

As a result, overall energy consumption was 210.32 fJ per MAC with *N* = 50 when the supply voltage of the neuron part was 0.65 V. This implies that the energy efficiency is 237.74 TOPS/W (Tera-Operations Per Second per Watt). This efficiency is comparable to the state of the art of the analog MAC macros (Seo et al., 2022; Choi et al., 2023). We summarized the potentially achievable energy efficiency with the number of inputs increased to 256, as shown in Table 3.

**Table 3:** The energy consumption breakdown and the energy efficiency of one MAC computation.

**Item**	**Unit**	**Simulation results**		**Estimation results**	
Number of inputs	–	50		100	256
**Energy consumption due to the axon line charge and discharge**					
Total capacitance of the AL per DL	fF	44		88	225.28
Supply voltage in synapse part	V	1.1			
**Energy consumption of the AL per DL**	fJ	53.24		106.48	272.59
**Energy consumption due to the dendrite line charge and discharge**					
Total capacitance of one DL	fF	895.44		1788.94	4576.66
Firing threshold of the neuron part	V	0.4	0.3	0.3	0.3
**Energy consumption of one DL**	fJ	143.27	80.59	161.00	411.90
**Energy consumption in the neuron part**					
Supply voltage in neuron part	V	0.75	0.65	0.65	0.65
**Energy consumption of one DL**	fJ	216.91	76.49	76.49	76.49
**Energy efficiency**					
Total energy consumption of one MAC	fJ	413.42	210.32	343.97	760.97
Energy/synapse operation	fJ	8.27	4.21	3.44	2.97
**Energy efficiency**	TOPS/W	120.94	237.74	290.72	336.41

Our purpose is to show the potential energy efficiency and computation precision of the TACT-based circuit, so we don't perform further design space exploration for optimizing the performance of the proposed circuit.

## 6 Discussion

We discuss some possible improvements on our PoC circuit design here.

In our design of the PoC circuit, we use a relatively long time window, i.e., 640 ns, to guarantee a moderate time resolution of 4–7 bits. The single MAC operation time is 1,300 ns, which consists of 20 ns for the neuron part reset and 640 ns for the input and output window, respectively. To improve the system latency, we can utilize massively parallel MAC operations to compensate for the relatively slow single MAC computation thanks to our simple readout circuit, which is area-efficient to make one column one readout possible, like in Khaddam-Aljameh et al. (2022) and Wan et al. (2022). At the system level, applying a pipeline scheme that uses the output timing window as the input window for subsequent computation can also be an effective approach Lim et al., (2020); Seo et al., (2022); Ambrogio et al., (2023).

We also designed a relatively large capacitance for the DL, which could degrade the area efficiency of the AIMC system. The total DL capacitance, *C*_*DL*_, is about 895 fF when the number of inputs is 50, as shown in Table 3. Because the wiring parasitic capacitance of the dendrite line per cell, *C*_*dl*_, is around 0.87 fF, an extra load capacitor, *C*_*l*_, will be about 850 fF, leading to area inefficiency in the neuron part. According to Equation 59, shortening the full-scale time window, decreasing the total integration current, or setting a higher *V*_*TH*_ will help minimize the capacitance to improve area efficiency. Regarding the decrease of the total integration current, we can make use of sparsity-aware optimization such as weight pruning. The sparsity, which we refer to as the ratio of zero weights to total weights, is typically 20%–50% Sze et al., (2017); Deng et al., (2020). Suppose the sparsity is 40%, then *C*_*DL*_ will be about 540 fF. To further minimize neuron part area overhead, we can also implement capacitors using a multi-layer metal-oxide-metal (MoM) structure lying on top of transistors in the synapse cell Valavi et al., (2019); Seo et al., (2022). Typically, the capacitance is 1–3 fF per cell area. By this means, *C*_*l*_ can be optimized to about 340 fF and such capacitance can be efficiently implemented by a MOSCAP Bavandpour et al., (2019a).

Shortening the full-scale time window also helps minimize *C*_*DL*_, but it will lead to a degradation of the computation precision. Because it involves improving the system latency and lowering the energy consumption of the neuron part, we are interested in estimating the results. When the number of inputs is increased to 256, the ENOB can be up to 5.4 bits, as shown in Table 2. If we keep the ENOB target as four bits, the full-scale time window can be shortened to about 250 ns. *C*_*DL*_ will be decreased to about 1,100 fF, and *C*_*l*_ can be minimized to a level under 100 fF, given that the weights' sparsity is 40%, *I*_*s*_ is 11.5 nA, and *V*_*TH*_ = 0.4 V. Accordingly, the energy consumption of the DL, *E*_*DL*_, and the neuron part, *E*_*NP*_, is optimized to about 176.6 and 29.9 fJ, respectively. This indicates an energy efficiency of 534.3 TOPS/W. We compare our work with the previous AIMC designs shown in Table 4. Our work shows a favorable performance.

**Table 4:** Performance summary and comparison with previous AIMC designs.

**Item**	**This work**		**Bavandpour et al. (2019a)**	**Lim et al. (2020)**	**Seo et al. (2022)**	**Wu et al. (2022)**	**Wan et al. (2022)**	**Le Gallo et al. (2023)**
Memory type	SRAM		NOR flash	SRAM	Analog memory	SRAM	RRAM	PCM
Technology	45 nm		55 nm	28 nm	28 nm	28 nm	130 nm	14 nm
Computing method	Time-domain & charge accumulation					Time-domain	Voltage	Current
**MVM core size [row**×**column], precision [bit], energy efficiency (EE) [TOPS/W], computation time [ns]**								
Core size	256 × 1	50 × 1	100 × 100/ 500 × 500	27 × 8	28 × 28	64 × 256	256 × 256	256 × 256
Input precision	Timing (ENB 4-6b)		Pulse width (ENB 6b)	Pulse width (ENB 3-7b)	Analog	4b/8b	4b	8b
Weight precision	1b/Analog		Analog	4b	5b	4b/8b	Analog	Analog
Output precision	Timing (ENB 4-6b)		Pulse width (ENB 6b)	Pulse width (analog)	Pulse width (analog)	Digital 14b/22b	Digital 6b	Digital 8b
EE [TOPS/W]	534.3 (4b)	237.7	85/135	7.1 (system)	332.7	85–112 (4b/4b/14b)	16	2.48
Computation time [ns]	500 (4b)	1,300	50–200	150*	740	105.6	4,000	520–1,518
Results	Estimated	Simulated	Simulated	Simulated	Measured	Measured	Measured	Measured
**Readout**								
Circuit	S-R Latch		TDC	Amp& Comparator	VTC	TDC	column ADC	CCO-based ADC
Compactness	○		○	×	○	○	○	○
Cycles or modulation steps for one MVM computation	Single		Single	Multiple	Multiple	Multiple	Multiple	Four
Processing w/o ADC	○		×	○	○	×	×	×
**Other features**								
Signed MAC	4-quadrant		2-quadrant	2-quadrant	2-quadrant	4-quadrant	4-quadrant	4-quadrant
PVT tolerance	○		○	×	○	NA	NA	NA

*Estimated value. **Colored as favorable features of this work.

When deploying DNNs to resource-constrained edge devices, trade-offs between accuracy, model size, latency, and energy efficiency need to be optimized, which is typically achieved by means of algorithm—hardware codesigns (Shuvo et al., 2022; Ngo et al., 2025).

Our future work includes the design and fabrication of a fully parallel MVM AIMC core or macro and the measurement of DNN inference accuracy, latency, energy efficiency on more realistic datasets such as CIFAR-10 and CIFAR-100. With respect to NN model optimization for the hardware, the improvement of the accuracy of the NN model without bias, discussed in Section 4.1, will also be an important effort.

## 7 Conclusions

We introduced a time-domain four-quadrant MAC calculation model where signed inputs are encoded using a differential pair of spikes, and signed weights are implemented through a dummy weights scheme. The output is represented by a pair of spikes, with their timing difference proportional to the MAC results, enabled by the added dummy weights. Since both inputs and outputs are encoded in a timing format, the AIMC core with this model can be seamlessly integrated with efficient DTCs and TDCs. We proposed architectures for our TACT-based MLP with the weights configured in a complementary scheme. We demonstrated a proof-of-concept (PoC) CMOS circuit equivalent to the previously proposed RC circuit, with preliminary simulation suggesting that the energy efficiency could reach hundreds of Tera Operations Per Second Per Watt (TOPS/W) and the precision could be four bit or higher.

Our proposed time-domain weighted-sum calculation model promises to be a suitable approach for intensive in-memory computing (IMC) of deep neural networks (DNNs) with moderate multi-bit inputs/outputs and weights, and avoiding or reducing the cost of ADC overhead so as to ultimately run the DNNs energy efficiently on edge devices for inference tasks.

## Data Availability

The original contributions presented in the study are included in the article/supplementary material, further inquiries can be directed to the corresponding author.

## References

[B1] Abas A. Bystrov A. Kinniment D. Maevsky O. Russell G. Yakovlev A.. (2002). Time difference amplifier. Electron. Lett. 38, 1437–1438. 10.1049/el:20020961

[B2] Aguirre F. Sebastian A. Le Gallo M. Song W. Wang T. Yang J. J.. (2024). Hardware implementation of memristor-based artificial neural networks. Nat. Commun. 15:1974. 10.1038/s41467-024-45670-9 38438350 PMC10912231

[B3] Al Maharmeh H. Ismail M. Alhawari M.. (2024). Energy-efficient time-domain computation for edge devices: challenges and prospects. Found. Trends Integr. Circuits Syst. 3, 1–50. 10.1561/3500000013

[B4] Al Maharmeh H. Sarhan N. J. Hung C.-C. Ismail M. Alhawari M. (2020). “Compute-in-time for deep neural network accelerators: challenges and prospects,” in 2020 IEEE 63rd International Midwest Symposium on Circuits and Systems (MWSCAS) (Springfield, MA: IEEE), 990–993. 10.1109/MWSCAS48704.2020.9184470

[B5] Al Maharmeh H. Sarhan N. J. Ismail M. Alhawari M. (2023). A 116 tops/w spatially unrolled time-domain accelerator utilizing laddered-inverter dtc for energy-efficient edge computing in 65 nm. IEEE Open J. Circuits Syst. 4, 308–323. 10.1109/OJCAS.2023.3332853

[B6] Ambrogio S. Narayanan P. Okazaki A. Fasoli A. Mackin C. Hosokawa K.. (2023). An analog-AI chip for energy-efficient speech recognition and transcription. Nature 620, 768–775. 10.1038/s41586-023-06337-5 37612392 PMC10447234

[B7] Asada K. Nakura T. Iizuka T. Ikeda M. (2018). Time-domain approach for analog circuits in deep sub-micron LSI. IEICE Electron. Express 15:20182001. 10.1587/elex.15.20182001

[B8] Bavandpour M. Mahmoodi M. R. Strukov D. B. (2019a). Energy-efficient time-domain vector-by-matrix multiplier for neurocomputing and beyond. IEEE Trans. Circuits Syst. II: Express Briefs 66, 1512–1516. 10.1109/TCSII.2019.2891688

[B9] Bavandpour M. Mahmoodi M. R. Strukov D. B. (2020). Acortex: an energy-efficient multipurpose mixed-signal inference accelerator. *IEEE J. Explor*. Solid-State Comput. Devices Circuits 6, 98–106. 10.1109/JXCDC.2020.2999581

[B10] Bavandpour M. Sahay S. Mahmoodi M. R. Strukov D. (2019b). Efficient mixed-signal neurocomputing via successive integration and rescaling. IEEE Trans Very Large Scale Integr. Syst. 28, 823–827. 10.1109/TVLSI.2019.2946516

[B11] Bavandpour M. Sahay S. Mahmoodi M. R. Strukov D. B. (2021). 3D-acortex: an ultra-compact energy-efficient neurocomputing platform based on commercial 3D-nand flash memories. Neuromorphic Comput. Eng. 1:014001. 10.1088/2634-4386/ac0775

[B12] Chen Y. Xie Y. Song L. Chen F. Tang T. (2020). A survey of accelerator architectures for deep neural networks. Engineering 6, 264–274. 10.1016/j.eng.2020.01.007

[B13] Chen Z. Jin Q. Yu Z. Wang Y. Yang K. (2022). “DCT-RAM: a driver-free process-in-memory 8t sram macro with multi-bit charge-domain computation and time-domain quantization,” in 2022 IEEE Custom Integrated Circuits Conference (CICC) (Newport Beach, CA: IEEE), 1–2. 10.1109/CICC53496.2022.9772826

[B14] Choi E. Choi I. Lukito V. Choi D.-H. Yi D. Chang I.-J.. (2023). “A 333tops/w logic-compatible multi-level embedded flash compute-in-memory macro with dual-slope computation,” in 2023 IEEE Custom Integrated Circuits Conference (CICC) (San Antonio, TX: IEEE), 1–2. 10.1109/CICC57935.2023.10121209

[B15] Choi J. Wang Z. Venkataramani S. Chuang P. I.-J. Srinivasan V. Gopalakrishnan K.. (2018). Pact: parameterized clipping activation for quantized neural networks. arXiv [Preprint]. arXiv:1805.06085. 10.48550/arXiv.1805.06085

[B16] Cireşan D. C. Meier U. Gambardella L. M. Schmidhuber J. (2010). Deep, big, simple neural nets for handwritten digit recognition. Neural Comput. 22, 3207–3220. 10.1162/NECO_a_00052 20858131

[B17] Courbariaux M. Bengio Y. David J.-P. (2015). “Binaryconnect: training deep neural networks with binary weights during propagations,” in Advances in Neural Information Processing System, 28 (Red Hook, NY: Curran Associates).

[B18] Deng L. Li G. Han S. Shi L. Xie Y. (2020). Model compression and hardware acceleration for neural networks: a comprehensive survey. Proc. IEEE 108, 485–532. 10.1109/JPROC.2020.2976475

[B19] Fick L. Blaauw D. Sylvester D. Skrzyniarz S. Parikh M. Fick D.. (2017). “Analog in-memory subthreshold deep neural network accelerator,” in 2017 IEEE Custom Integrated Circuits Conference (CICC) (Austin, TX: IEEE), 1–4. 10.1109/CICC.2017.7993629

[B20] Fick L. Skrzyniarz S. Parikh M. Henry M. B. Fick D. (2022). “Analog matrix processor for edge AI real-time video analytics.” in 2*022 IEEE International Solid-State Circuits Conference (ISSCC), Vol. 65* (San Francisco, CA: IEEE), 260–262. 10.1109/ISSCC42614.2022.9731773

[B21] Freye F. Lou J. Bengel C. Menzel S. Wiefels S. Gemmeke T.. (2022). Memristive devices for time domain compute-in-memory. *IEEE J. Explor*. Solid-State Comput. Devices Circuits 8, 119–127. 10.1109/JXCDC.2022.3217098

[B22] Freye F. Lou J. Lanius C. Gemmeke T. (2024). “Merits of time-domain computing for vmm-a quantitative comparison,” in 2024 25th International Symposium on Quality Electronic Design (ISQED) (San Francisco, CA: IEEE), 1–8. 10.1109/ISQED60706.2024.10528682

[B23] Gonugondla S. K. Sakr C. Dbouk H. Shanbhag N. R. (2021). Fundamental limits on energy-delay-accuracy of in-memory architectures in inference applications. *IEEE Trans. Comput.-Aided Des. Integr*. Circuits Syst. 41, 3188–3201. 10.1109/TCAD.2021.3124757

[B24] Guo X. Bayat F. M. Bavandpour M. Klachko M. Mahmoodi M. Prezioso M.. (2017). “Fast, energy-efficient, robust, and reproducible mixed-signal neuromorphic classifier based on embedded nor flash memory technology,” in 2017 IEEE International Electron Devices Meeting (IEDM) (San Francisco, CA: IEEE), 6–5. 10.1109/IEDM.2017.8268341

[B25] Gupta S. Agrawal A. Gopalakrishnan K. Narayanan P. (2015). “Deep learning with limited numerical precision,” in International Conference on Machine Learning (Lille: JMLR.org), 1737–1746.

[B26] Hasler J. Marr B. (2013). Finding a roadmap to achieve large neuromorphic hardware systems. Front. Neurosci. 7:118. 10.3389/fnins.2013.00118 24058330 PMC3767911

[B27] Horowitz M. (2014). “1.1 computing's energy problem (and what we can do about it),” in 2014 IEEE international solid-state circuits conference digest of technical papers (ISSCC) (San Francisco, CA: IEEE), 10–14. 10.1109/ISSCC.2014.6757323

[B28] Jia H. Ozatay M. Tang Y. Valavi H. Pathak R. Lee J.. (2021a). “15.1 a programmable neural-network inference accelerator based on scalable in-memory computing,” in 2021 IEEE International Solid-State Circuits Conference (ISSCC), Vol. 64 (San Francisco, CA: IEEE), 236–238. 10.1109/ISSCC42613.2021.9365788

[B29] Jia H. Ozatay M. Tang Y. Valavi H. Pathak R. Lee J.. (2021b). Scalable and programmable neural network inference accelerator based on in-memory computing. IEEE J. Solid-State Circuits 57, 198–211. 10.1109/JSSC.2021.3119018

[B30] Jiang H. Huang S. Li W. Yu S. (2022). Enna: An efficient neural network accelerator design based on adc-free compute-in-memory subarrays. IEEE Trans. Circuits Syst. I: Regul. Papers 70, 353–363. 10.1109/TCSI.2022.3208755

[B31] Jiang Z. Yin S. Seo J.-S. Seok M. (2020). C3sram: an in-memory-computing sram macro based on robust capacitive coupling computing mechanism. IEEE J. Solid-State Circuits 55, 1888–1897. 10.1109/JSSC.2020.2992886

[B32] Joshi V. Le Gallo M. Haefeli S. Boybat I. Nandakumar S. R. Piveteau C.. (2020). Accurate deep neural network inference using computational phase-change memory. Nat. Commun. 11:2473. 10.1038/s41467-020-16108-9 32424184 PMC7235046

[B33] Khaddam-Aljameh R. Stanisavljevic M. Mas J. F. Karunaratne G. Brändli M. Liu F.. (2022). Hermes-core—a 1.59-tops/mm 2 pcm on 14-nm cmos in-memory compute core using 300-ps/lsb linearized cco-based adcs. IEEE J. Solid-State Circuits 57, 1027–1038. 10.1109/JSSC.2022.3140414

[B34] Kingra S. K. Parmar V. Sharma M. Suri M. (2022). Time-multiplexed in-memory computation scheme for mapping quantized neural networks on hybrid cmos-oxram building blocks. IEEE Trans. Nanotechnol. 21, 406–412. 10.1109/TNANO.2022.3193921

[B35] Kneip A. Bol D. (2021). Impact of analog non-idealities on the design space of 6t-sram current-domain dot-product operators for in-memory computing. IEEE Trans. Circuits Sys. I: Regul. Papers 68, 1931–1944. 10.1109/TCSI.2021.3058510

[B36] Krizhevsky A. Sutskever I. Hinton G. E. (2012). “Imagenet classification with deep convolutional neural networks,” in Advances in Neural Information Processing System (Red Hook, NY: Curran Associates), 25.

[B37] Le Gallo M. Hrynkevych O. Kersting B. Karunaratne G. Vasilopoulos A. Khaddam-Aljameh R.. (2024). Demonstration of 4-quadrant analog in-memory matrix multiplication in a single modulation. Npj Unconv. Comput. 1:11. 10.1038/s44335-024-00010-4 39372606 PMC11449787

[B38] Le Gallo M. Khaddam-Aljameh R. Stanisavljevic M. Vasilopoulos A. Kersting B. Dazzi M.. (2023). A 64-core mixed-signal in-memory compute chip based on phase-change memory for deep neural network inference. Nat. Electron. 6, 680–693. 10.1038/s41928-023-01010-1

[B39] LeCun Y. Bengio Y. Hinton G. (2015). Deep learning. Nature 521, 436–444. 10.1038/nature14539 26017442

[B40] LeCun Y. Bottou L. Bengio Y. Haffner P. (2002). Gradient-based learning applied to document recognition. Proc. IEEE 86, 2278–2324. 10.1109/5.726791

[B41] Lim J. Choi M. Liu B. Kang T. Li Z. Wang Z.. (2020). “AA-ResNet: energy efficient all-analog resnet accelerator,” in 2020 IEEE 63rd International Midwest Symposium on Circuits and Systems (MWSCAS) (Springfield, MA: IEEE), 603–606. 10.1109/MWSCAS48704.2020.9184587

[B42] Maass W. (1997a). Fast sigmoidal networks via spiking neurons. Neural Comput. 9, 279–304. 10.1162/neco.1997.9.2.279 9117904

[B43] Maass W. (1997b). Networks of spiking neurons: the third generation of neural network models. Neural Netw. 10, 1659–1671. 10.1016/S0893-6080(97)00011-7

[B44] Maass W. (1999). Computing with spiking neurons. Pulsed Neural Netw. 2, 55–85. 10.7551/mitpress/5704.003.0006

[B45] Mahmoodi M. R. Strukov D. (2018). “An ultra-low energy internally analog, externally digital vector-matrix multiplier based on nor flash memory technology,” in Proceedings of the 55th Annual Design Automation Conference (New York, NY: ACM), 1–6. 10.1145/3195970.3195989

[B46] Marinella M. J. Agarwal S. Hsia A. Richter I. Jacobs-Gedrim R. Niroula J.. (2018). Multiscale co-design analysis of energy, latency, area, and accuracy of a reram analog neural training accelerator. IEEE J. Emerg. Sel. Top. Circuits Syst. 8, 86–101. 10.1109/JETCAS.2018.2796379

[B47] McKinstry J. L. Esser S. K. Appuswamy R. Bablani D. Arthur J. V. Yildiz I. B.. (2018). Discovering low-precision networks close to full-precision networks for efficient embedded inference. arXiv [preprint] arXiv:1809.04191. 10.48550/arXiv.1809.04191

[B48] Morie T. Liang H. Tohara T. Tanaka H. Igarashi M. Samukawa S.. (2016). “Spike-based time-domain weighted-sum calculation using nanodevices for low power operation,” in 2016 IEEE 16th International Conference on Nanotechnology (IEEE-NANO) (Sendai: IEEE), 390–392. 10.1109/NANO.2016.7751490

[B49] Morie T. Sun Y. Liang H. Igarashi M. Huang C.-H. Samukawa S.. (2010). “A 2-dimensional si nanodisk array structure for spiking neuron models,” in Proceedings of 2010 IEEE International Symposium on Circuits and Systems (Paris: IEEE), 781–784. 10.1109/ISCAS.2010.5537456

[B50] Nägele R. Finkbeiner J. Stadtlander V. Grözing M. Berroth M. (2023). Analog multiply-accumulate cell with multi-bit resolution for all-analog AI inference accelerators. IEEE Trans. Circuits Syst. I: Regul. Papers 70, 3509–3521. 10.1109/TCSI.2023.3268728

[B51] Nair V. Hinton G. E. (2010). “Rectified linear units improve restricted Boltzmann machines,” in Proceedings of the 27th International Conference on Machine Learning (ICML-10) (Madison, WI: Omnipress), 807–814.

[B52] Narayanan P. Ambrogio S. Okazaki A. Hosokawa K. Tsai H. Nomura A.. (2021). Fully on-chip mac at 14 nm enabled by accurate row-wise programming of pcm-based weights and parallel vector-transport in duration-format. IEEE Trans. Electron Devices 68, 6629–6636. 10.1109/TED.2021.3115993

[B53] Ngo D. Park H.-C. Kang B. (2025). Edge intelligence: a review of deep neural network inference in resource-limited environments. Electronics 14:2495. 10.3390/electronics14122495

[B54] Prezioso M. Merrikh-Bayat F. Hoskins B. D. Adam G. C. Likharev K. K. Strukov D. B.. (2015). Training and operation of an integrated neuromorphic network based on metal-oxide memristors. Nature 521, 61–64. 10.1038/nature14441 25951284

[B55] Roy K. Jaiswal A. Panda P. (2019). Towards spike-based machine intelligence with neuromorphic computing. Nature 575, 607–617. 10.1038/s41586-019-1677-2 31776490

[B56] Sahay S. Bavandpour M. Mahmoodi M. R. Strukov D. (2020). Energy-efficient moderate precision time-domain mixed-signal vector-by-matrix multiplier exploiting 1t-1r arrays. IEEE J. Explor. Solid-State Comput. Devices Circuits 6, 18–26. 10.1109/JXCDC.2020.2981048

[B57] Schlottmann C. R. Hasler P. E. (2011). A highly dense, low power, programmable analog vector-matrix multiplier: The FPAA implementation. IEEE J. Emer. Sel. Top. Circuits Syst. 1, 403–411. 10.1109/JETCAS.2011.2165755

[B58] Sebastian A. Le Gallo M. Khaddam-Aljameh R. Eleftheriou E. (2020). Memory devices and applications for in-memory computing. Nat. Nanotechnol. 15, 529–544. 10.1038/s41565-020-0655-z 32231270

[B59] Seo J.-O. Seok M. Cho S. (2022). “Archon: a 332.7 tops/w 5b variation-tolerant analog cnn processor featuring analog neuronal computation unit and analog memory,” in 2022 IEEE International Solid-State Circuits Conference (ISSCC), Volume 65 (San Francisco, CA: IEEE), 258–260. 10.1109/ISSCC42614.2022.9731654

[B60] Shafiee A. Nag A. Muralimanohar N. Balasubramonian R. Strachan J. P. Hu M.. (2016). ISAAC: a convolutional neural network accelerator with in-situ analog arithmetic in crossbars. ACM SIGARCH Comput. Archit. News 44, 14–26. 10.1145/3007787.3001139

[B61] Shukla S. Fleischer B. Ziegler M. Silberman J. Oh J. Srinivasan V.. (2019). A scalable multi-teraops core for AI training and inference. IEEE Solid-State Circuits Lett. 1, 217–220. 10.1109/LSSC.2019.2902738

[B62] Shuvo M. M. H. Islam S. K. Cheng J. Morshed B. I. (2022). Efficient acceleration of deep learning inference on resource-constrained edge devices: a review. Proc. IEEE 111, 42–91. 10.1109/JPROC.2022.3226481

[B63] Sze V. Chen Y.-H. Yang T.-J. Emer J. S. (2017). Efficient processing of deep neural networks: a tutorial and survey. Proc. IEEE 105, 2295–2329. 10.1109/JPROC.2017.2761740

[B64] Tohara T. Liang H. Tanaka H. Igarashi M. Samukawa S. Endo K.. (2016). Silicon nanodisk array with a fin field-effect transistor for time-domain weighted sum calculation toward massively parallel spiking neural networks. Appl. Phys. Express 9:034201. 10.7567/APEX.9.034201

[B65] Tsai H. Ambrogio S. Narayanan P. Shelby R. M. Burr G. W. (2018). Recent progress in analog memory-based accelerators for deep learning. J. Phys. D Appl. Phys. 51:283001. 10.1088/1361-6463/aac8a5

[B66] Valavi H. Ramadge P. J. Nestler E. Verma N. (2019). A 64-tile 2.4-mb in-memory-computing cnn accelerator employing charge-domain compute. IEEE J. Solid-State Circuits 54, 1789–1799. 10.1109/JSSC.2019.2899730

[B67] Verma N. Jia H. Valavi H. Tang Y. Ozatay M. Chen L.-Y.. (2019). In-memory computing: advances and prospects. IEEE Solid-State Circuits Mag. 11, 43–55. 10.1109/MSSC.2019.2922889

[B68] Wan W. Kubendran R. Schaefer C. Eryilmaz S. B. Zhang W. Wu D.. (2022). A compute-in-memory chip based on resistive random-access memory. Nature 608, 504–512. 10.1038/s41586-022-04992-8 35978128 PMC9385482

[B69] Wang L. Ye W. Dou C. Si X. Xu X. Liu J.. (2021). Efficient and robust nonvolatile computing-in-memory based on voltage division in 2t2r rram with input-dependent sensing control. IEEE Trans. Circuits Syst. II: Express Briefs 68, 1640–1644. 10.1109/TCSII.2021.3067385

[B70] Wang Q. Tamukoh H. Morie T. (2016). “Time-domain weighted-sum calculation for ultimately low power vlsi neural networks,” in International Conference on Neural Information Processing (Cham: Springer), 240–247. 10.1007/978-3-319-46687-3_26

[B71] Wang Q. Tamukoh H. Morie T. (2018). A time-domain analog weighted-sum calculation model for extremely low power vlsi implementation of multi-layer neural networks. arXiv [preprint]. arXiv:1810.06819. 10.48550/arXiv:1810.06819 PMC12463932 41017977

[B72] Wang S. Zhou T. Bilmes J. (2019). “Bias also matters: bias attribution for deep neural network explanation,” in International Conference on Machine Learning (Long Beach, CA), 6659–6667.

[B73] Wu P.-C. Su J.-W. Chung Y.-L. Hong L.-Y. Ren J.-S. Chang F.-C.. (2022). “A 28nm 1mb time-domain computing-in-memory 6t-sram macro with a 6.6 ns latency, 1241gops and 37.01 tops/w for 8b-mac operations for edge-AI devices,” in 2022 IEEE International Solid-State Circuits Conference (ISSCC), Volume 65 (San Francisco, CA: IEEE), 1–3. 10.1109/ISSCC42614.2022.9731681

[B74] Xiao T. P. Feinberg B. Bennett C. H. Prabhakar V. Saxena P. Agrawal V.. (2023). On the accuracy of analog neural network inference accelerators. IEEE Circuits Syst. Mag. 22, 26–48. 10.1109/MCAS.2022.3214409

[B75] Yamaguchi M. Iwamoto G. Nishimura Y. Tamukoh H. Morie T. (2020). An energy-efficient time-domain analog cmos binaryconnect neural network processor based on a pulse-width modulation approach. IEEE Access 9, 2644–2654. 10.1109/ACCESS.2020.3047619

[B76] Yang J. Kong Y. Wang Z. Liu Y. Wang B. Yin S.. (2019). “24.4 sandwich-ram: an energy-efficient in-memory bwn architecture with pulse-width modulation,” in 2019 IEEE International Solid-State Circuits Conference-(ISSCC) (San Francisco, CA: IEEE), 394–396. 10.1109/ISSCC.2019.8662435

[B77] Zhang M. Wang J. Wu J. Belatreche A. Amornpaisannon B. Zhang Z.. (2021). Rectified linear postsynaptic potential function for backpropagation in deep spiking neural networks. IEEE Trans. Neural Netw. Learn. Syst. 33, 1947–1958. 10.1109/TNNLS.2021.3110991 34534091

